# Influence of Intermittent Cold Stimulations on CREB and Its Targeting Genes in Muscle: Investigations into Molecular Mechanisms of Local Cryotherapy

**DOI:** 10.3390/ijms21134588

**Published:** 2020-06-28

**Authors:** Takehito Sugasawa, Yoshiya Tome, Yoshinori Takeuchi, Yasuko Yoshida, Naoya Yahagi, Rahul Sharma, Yuichi Aita, Haruna Ueda, Reina Maruyama, Kaoru Takeuchi, Shohei Morita, Yasushi Kawamai, Kazuhiro Takekoshi

**Affiliations:** 1 Laboratory of Laboratory/Sports Medicine, Division of Clinical Medicine, Faculty of Medicine, University of Tsukuba, 1-1-1 Tennodai, Tsukuba, Ibaraki 305-8577, Japan; y-kawa@md.tsukuba.ac.jp (Y.K.); k-takemd@md.tsukuba.ac.jp (K.T.); 2 Department of Medical Technology, Faculty of Health Sciences, Tsukuba International University, 6-20-1 Manabe, Tsuchiura, Ibaraki 300-0051, Japan; tome@tius.ac.jp (Y.T.); shinbelon@mwa.biglobe.ne.jp (Y.Y.); 3 Nutrigenomics Research Group, Faculty of Medicine, University of Tsukuba, 1-1-1 Tennodai, Tsukuba, Ibaraki 305-8575, Japan; yoshinori-takeuchi@umin.ac.jp (Y.T.); nyahagi-tky@umin.ac.jp (N.Y.); metabmetabmetab@gmail.com (Y.A.); 4 Department of Internal Medicine (Endocrinology and Metabolism), Faculty of Medicine, University of Tsukuba, 1-1-1 Tennodai, Tsukuba, Ibaraki 305-8575, Japan; rahul222ab@hotmail.com; 5 Master’s Program in Medical Sciences, Graduate School of Comprehensive Human Sciences, University of Tsukuba, 1-1-1 Tennodai, Tsukuba, Ibaraki 305-8577, Japan; s1821255@s.tsukuba.ac.jp (H.U.); s1721322@s.tsukuba.ac.jp (R.M.); 6 Laboratory of Environmental Microbiology, Division of Basic Medicine, Faculty of Medicine, University of Tsukuba, 1-1-1 Tennodai, Tsukuba, Ibaraki 305-8575, Japan; ktakeuch@md.tsukuba.ac.jp; 7 Cluster of Medical Sciences, School of Medicine, University of Tsukuba, 1-1-1 Tennodai, Tsukuba, Ibaraki 305-8575, Japan; moris.swtmed@gmail.com

**Keywords:** CREB, cryotherapy, gene expression, icing, mitochondria, Pgc-1α, transcription

## Abstract

Local cryotherapy is widely used as a treatment for sports-related skeletal muscle injuries. The molecular mechanisms are unknown. To clarify these mechanisms, we applied one to three 15-min cold stimulations at 4 °C to various cell lines (in vitro), the tibialis anterior (TA) muscle (ex vivo), and mouse limbs (in vivo). In the in vitro assay, cyclic AMP (cAMP) response element binding protein 1 (CREB1) was markedly phosphorylated (p-CREB1), and the CREB-binding protein (CBP) was recruited to p-CREB-1 in response to two or three cold stimulations. In a reporter assay with the cAMP-responsive element, the signals significantly increased after two to three cold stimulations at 4 °C. In the ex vivo study, CREB-targeting genes were significantly upregulated following two or three cold stimulations. The in vivo experiment disclosed that cold stimulation of a mouse limb for 9 days significantly increased mitochondrial DNA copy number and upregulated genes involved in mitochondrial biogenesis. The results suggest that local cryotherapy increases CREB transcription and upregulates CREB-targeting genes, in a manner dependent on cold stimulation frequency and duration. This information will inform further investigations into local cryotherapy as a treatment for sports-related skeletal muscle trauma.

## 1. Introduction

Local cryotherapy has been widely used in the acute treatment of traumatic injuries, such as fractures, dislocations, sprains, and pulled muscles. It has also been applied for recovery after daily training and sports competitions. At all levels of athletic performance, it is common to see ice bags applied to knees, shoulder joints, thigh muscles, etc. The use of cryotherapy in the management of sports injuries was first reported in Greece in the 1950s. Knight proposed that cryotherapy retards cellular metabolism and mitigates impairment caused by secondary hypoxia in the injured area [1]. This theory has been accepted as the standard explanation for the mechanism underlying local cryotherapy in sports injury management. However, there are numerous uncertainties regarding the efficacy and basic mechanism of local cryotherapy, as the previous study described below.

The previous in vivo studies reported that cryotherapy was both effective [2,3,4] and ineffective [5,6] in the treatment of soft tissue injuries. Moreover, two systematic reviews on cryotherapy for soft tissue injuries concluded that the efficacy of cryotherapy remains unclear, and further study is required to elucidate its mechanism [7,8]. On the other hand, Hohenauer et al. [9] presented a meta-analysis of the effects of post-exercise cryotherapy on recovery on humans. In the results, cold water immersions after exercise significantly improved delayed-onset muscle soreness (DOMS) and ratings of perceived exertion (RPE), relative to the untreated control [9]. Moreover, we also previously conducted experiments using cells lines. In the results, three intermittent cold stimulations at 4 °C and 17 °C increase mitochondrial number and activity, suggesting this response is temperature-dependent, and 4 to 17 °C is effective temperature [10]. As the above-mentioned study reports, the clear effects and efficacies of the cryotherapy are still unknown until today. To solve these problems, elucidations of the molecular mechanisms are an important. Therefore, we aimed to elucidate the basic molecular mechanisms of local cryotherapy (especially transcription and gene expression) in this study.

Our previous study revealed that cold stimulation on three times at the optimal temperature increases mitochondrial number and activity [10]. Additionally, it was reported that cyclic AMP (cAMP) response element binding protein 1 (CREB1) as a transcriptional factor is associated with mitochondrial biogenesis via the upregulation of peroxisome proliferator-activated receptor gamma coactivator 1-alpha (PGC-1α) as a CREB-targeting gene [11,12,13]. Therefore, we especially focused on the CREB1 activity, and it was hypothesized that multiple cold stimulations at the optimal temperature enhance mitochondrial biogenesis through transcriptional CREB activation and *Pgc-1α* upregulation.

## 2. Results

### 2.1. Cold Stimulation Induced CREB1 Phosphorylation in a Frequency- and Duration-Dependent Manner In Vitro

**Figure 1:**
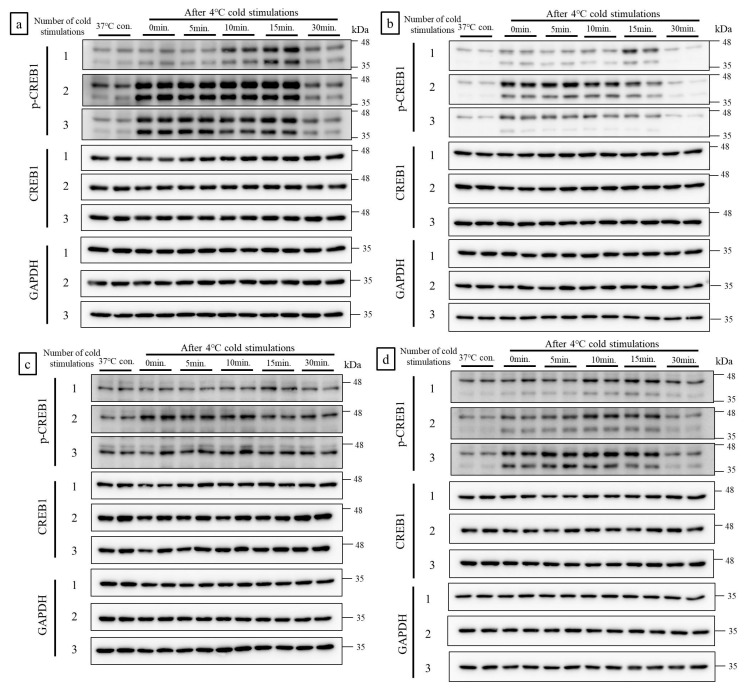
Cold stimulation induced cyclic AMP (cAMP) response element binding protein 1 (CREB1) phosphorylation in a frequency- and duration-dependent manner in vitro. In four cell lines of C2C12, 3T3-L1, human fibroblast (HF) and L6, cold stimulation induced CREB1 phosphorylation in a Figure 1. phosphorylation, whereas two and three cold stimulations markedly induced CREB phosphorylation. The strongest and weakest CREB1 phosphorylation was apparent in the C2C12 and L6 cell lines, respectively. (**a**): C2C12 cells; (**b**): 3T3-L1 cells; (**c**): L6 cells; (**d**): HF cells. Con.: Control (no cold stimulation).

Two bands appeared in western blot (WB) analysis of C2C12 (mouse myoblast), 3T3-L1 (mouse fibroblast), and human fibroblast (HF) cells that make up muscle tissue, in response to anti phosphorylated CREB1 (p-CREB1) antibody. The upper band was p-CREB1 and the lower band was phosphorylated cyclic AMP-dependent transcription factor 1 (p-ATF1). Both of these have the same function. In four cell lines of C2C12, 3T3-L1, HF, and L6 (rat myoblast), cold stimulation induced CREB1 phosphorylation in a frequency- and duration-dependent manner. A single cold stimulation slightly induced CREB1 phosphorylation, whereas two and three cold stimulations markedly induced CREB phosphorylation. The strongest and weakest CREB1 phosphorylation was apparent in the C2C12 and L6 cell lines, respectively. Thirty minutes after the final cold stimulation, the p-CREB1 bands in all cell lines returned to the same intensity as those of the control (Figure 1a–d).

### 2.2. Cold Stimulation Did Not Cause Cell Damage In Vitro

**Figure 2:**
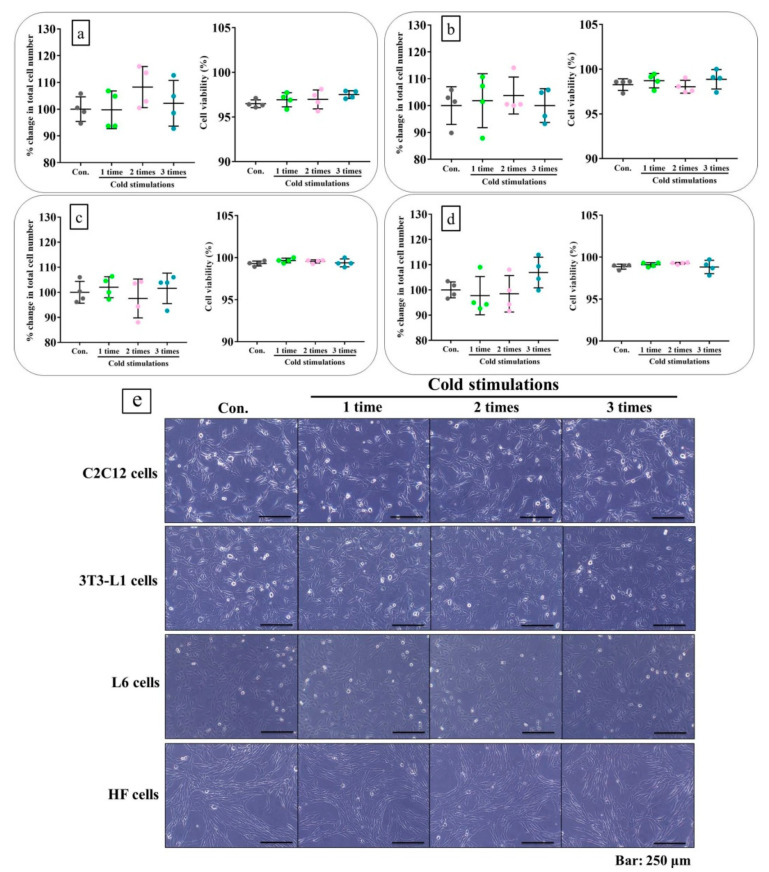
Cold stimulation did not cause cell damage in vitro. The figures of (**a**–**d**) show the evaluation of percent change in total cell number and viability using trypan blue assay on each cell line. In the result, no cell damages were observed. The figure of e shows the evaluation of cell morphologies, which were also observed no cell damages. (**a**): C2C12 cells; (**b**): 3T3-L1 cells; (**c**): L6 cells; (**d**): HF cells. (**e**): cell morphology. Con: control (no cold stimulation).

Total cell numbers, viability, and morphology served as indices of damage in the four cell lines made up muscle tissue. No changes relative to the control were detected, even after three cold stimulations (Figure 2a–e).

### 2.3. CREB-Binding Protein (CBP) Was Recruited to p-CREB in Response to Cold Stimulation In Vitro

**Figure 3:**
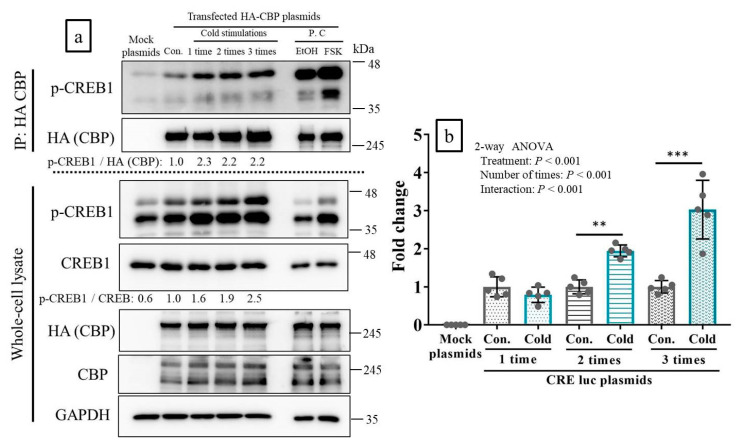
Cold stimulation induced recruitment of CREB-binding protein (CBP) to phosphorylated-CREB1 (p-CREB1) and activated CREB transcription in vitro. (**a**): Immunoprecipitation and western blot (WB) analyses to assess for the recruitment of CBP to p-CREB1 upon cold stimulations. P.C lanes show positive control with forskolin (FSK) or ethanol (EtOH), to confirm the association of CBP with p-CREB. Mock plasmids were used as negative controls, and cAMP response element (CRE)- luciferase (luc) plasmids were used to measure the transcriptional activities of CREB. (**b**): Reporter assay to analyze the transcriptional activities of CREB upon cold stimulations using CRE-luc plasmids (*n* = 5 per group). Mock plasmids were used as negative controls with values of approximately 0. Con: control (no cold-stimulation). Cold: 15-min cold-stimulations. ** *p* < 0.01, *** *p* < 0.001 according to Bonferroni correction as a post-hoc test.

Two bands appeared in response to anti p-CREP1 antibody. The upper band was p-CREB and the lower band was p-ATF1 on the human embryonic kidney (HEK) 293 cells. An interaction between the CREB-binding protein (CBP) and phosphorylated-CREB1 (p-CREB1) was observed in the control lane. The interaction was also strong in response to the cold stimulations and induced an increase in CREB1 phosphorylation (Figure 3a, lane: 1–3 cold stimulations). Therefore, CBP recruitment to p-CREB1 increased in response to cold stimulation. The p-CREB1:CREB1 ratios in whole cell lysates increased with the number of cold stimulations. Nevertheless, the p-CREB1: HA (CBP) ratios (indices of post-IP binding intensity) increased equally for all cold stimulation repetitions (Figure 3a).

### 2.4. Cold Stimulation Activated CREB Transcription In Vitro

Reporter assay involving a luciferase (luc) expression plasmid with cAMP response element (CRE)-driven transcription revealed that cold stimulation significantly activated CREB transcription, in correlation with stimulation frequency. Unlike a single cold stimulation, two or three cold stimulations activated CREB-mediated transcription. Three cold stimulations induced the strongest transcriptional activity (Figure 3b).

### 2.5. Cold Stimulations Upregulated the CREB-Targeting Gene and Induced CREB1 Phosphorylation in Skeltal Muscle Ex Vivo

**Figure 4:**
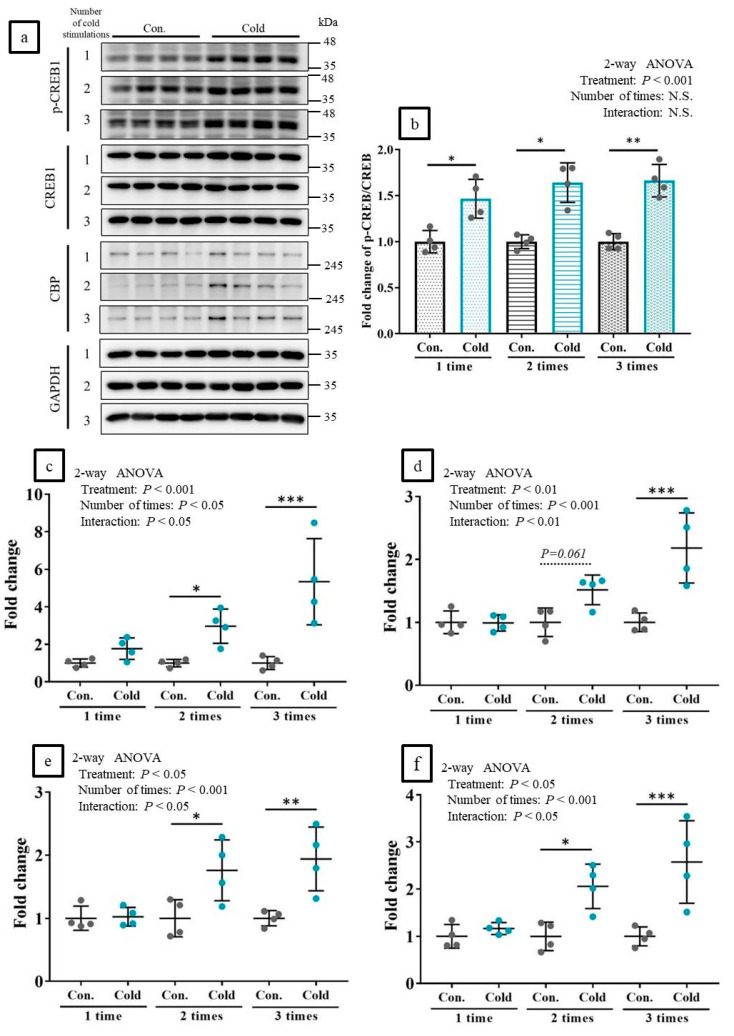
Cold stimulations significantly induced CREB1 phosphorylation and upregulated the target genes of CREB ex vivo. (**a**): Western blot analysis of p-CREB1 and other factors. (**b**): Quantification of the band intensities of p-CREB1/CREB1 on the western blots (*n* = four per group). (**c**–**f**): Expressions levels of CREB-targeted genes upon cold stimulations (*n* = four per group). (**c**–**f**): *Pgc-1α*, *Creb1*, *Glut4*, and *Cpt1b*, respectively. Con: Control (no cold-stimulation). Cold: 15-min cold-stimulations. * *p* < 0.05, ** *p* < 0.01, *** *p* < 0.001 according to Bonferroni correction as a post-hoc test. N.S.: not significant.

One to three cold stimulations significantly induced CREB1 phosphorylation in tibialis anterior (TA) muscle on ex vivo experiments. However, the intensities of the p-CREB1 bands in response to one, two, or three cold stimulations were nearly equal (Figure 4a,b). Conversely, the expression levels of the CREB1-targeting genes significantly increased with the number of cold stimulations. A single cold stimulation had almost no effect at enhancing gene expression, whereas two or three cold stimulations (especially the latter) significantly upregulated the genes (Figure 4c–f).

### 2.6. Cold Stimulations Also Upregulated the CREB-Targeting Gene and Induced CREB1 Phosphorylation on Stkeltal Muscle In Vivo

**Figure 5:**
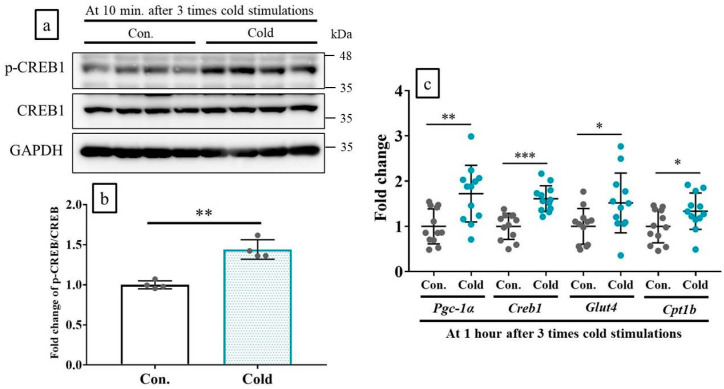
Cold stimulations significantly induce CREB1 phosphorylation and upregulated CREB1-targeting genes in vivo. (**a**): WB analysis of p-CREB1 and other factors; (**b**): quantification of band intensities of p-CREB1/CREB1 (*n* = 4 per group); (**c**): the expression of CREB-targeting genes (*n* = 12 per group). * *p* < 0.05, ** *p* < 0.01, *** *p* < 0.001 according to Welch’s *t*-test.

Ten minutes following the third cold stimulation, CREB1 phosphorylation was significantly induced in the TA muscle (Figure 5a,b). By 1 h, the CREB1-targeting genes were also significantly upregulated (Figure 5c).

### 2.7. Chronic Cold Stimulation Increased Mitochondrial DNA Copy Number, Mitochondrial Biogenesis, and Its Component Gene Expression Levels in the Skeletal Muscle, Whereas Acute Cold Stimulation Had No Such Effects

**Figure 6:**
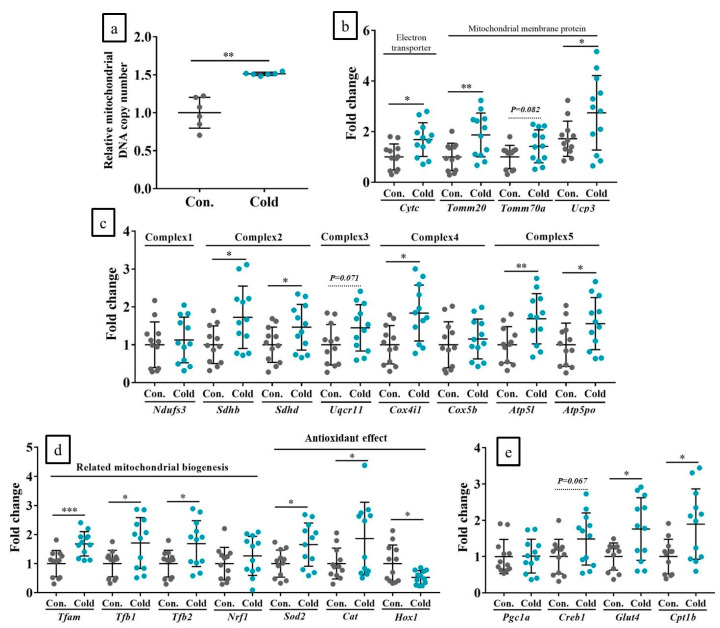
Chronic cold stimulation increased mitochondrial DNA (mtDNA) copy number and mitochondrial biogenesis in vivo. (**a**): Mitochondrial DNA copy number; (**b**): mitochondrial component genes; (**c**): mitochondrial complex genes; (**d**): *Pgc1-α* regulated genes; (**e**): CREB-targeting genes in response to chronic in vivo cold stimulation. Con: Control (no cold stimulation); Cold: 15-min cold stimulations. *n* = 12 per group. * *p* < 0.05, ** *p* < 0.01, *** *p* < 0.001 according to Welch’s *t*-test.

Chronic cold stimulations of mouse limb significantly increased mitochondrial DNA (mtDNA) copy number in the muscles. Moreover, the gene expression level about mitochondrial components, complexes, and biogenesis and the expression levels of CREB-1 targeting genes were also increased. However, the gene expressions of *Pgc-1a* were unchanged. The average rate of increase was approximately 1.5–2× (Figure 6a–e). However, acute cold stimulations did not have any of these effects with *Ndufs1* (a complex 1 gene), which was upregulated by only approximately 4× (Supplemental Figure S2a–e).

### 2.8. Cold Stimulation Had No Adverse Effect

**Figure 7:**
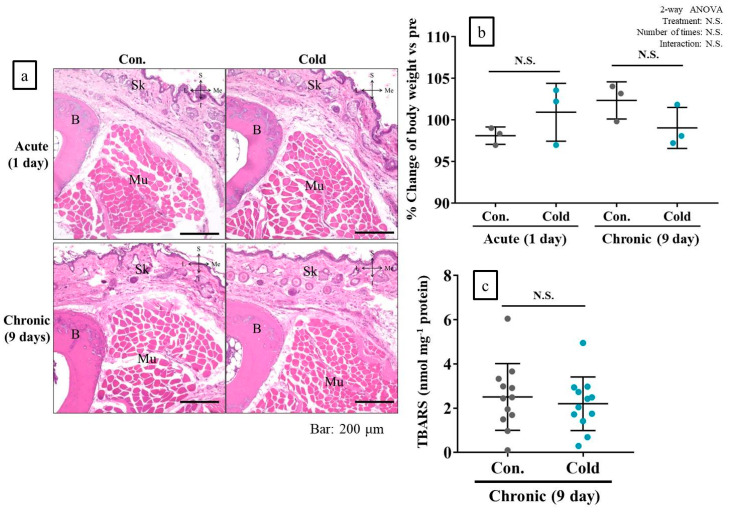
Acute and chronic cold stimulations did not induce adverse effects in muscle, bone, or skin tissue, and did not alter body weight in vivo. (**a**): Representative hematoxylin and eosin (H&E)-stained tissue sample; (**b**): % change in body weight relative to pre-experiment; (**c**): thiobarbituric acid reactive substances (TBARS) levels in limb muscle in response to chronic cold stimulation. B: Bone (second metatarsal); Sk: Skin; Mu: muscle (first dorsal interosseous muscles of foot); S: Superior; I: Inferior; L: Lateral; Me: Medial. Con: Control (no cold stimulation); Cold: 15-min cold stimulation. *n* = 3 for body weight and *n* = 12 for TBARS levels. N.S.: Not significant.

In hematoxylin and eosin (H&E)-stained specimens of mouse feet subjected to acute and chronic cold stimulation, there was no evidence of any adverse effect, such as tissue inflammation or degeneration of muscle, bone, or skin tissue. Neither treatment altered body weight or muscle thiobarbituric acid reactive substances (TBARS) levels referring to oxidative stress (Figure 7a–c).

## 3. Discussion

In the three experiments (in vitro, ex vivo, and in vivo), cold stimulation induced CREB1 phosphorylation, even though the sympathetic nerves were not stimulated in the in vitro and ex vivo experiments. Moreover, the cold stimulations also activated CREB1 transcription. The upregulated CREB-targeting genes in both ex vivo and in vivo experiments, and the level of induced CREB1 phosphorylation, were almost the same between the ex vivo and in vivo experiments upon cold stimulations. In response to cold exposure, the sensory nerves in the peripheral tissues transduce the signal to the hypothalamus, which regulates the sympathetic nervous system (SNS), and triggers the release of noradrenaline (NE) from the nerve terminals and entry into the target tissue [14]. NE binds to the β-adrenergic receptor (β-AR), and activates downstream signals, including the production of cyclic AMP (cAMP), activation of protein kinase A (PKA), phosphorylation of CREB, upregulation of Pgc1-α, uncoupling of protein 1 (Ucp1), and other events [14]. In the present study, however, the SNS had no apparent influence on the release of NE to the β-AR, because the in vitro and ex vivo experiments did not consider the nervous systems. It is possible that cryotherapy directly induces CREB1 phosphorylation and CREB-targeting gene upregulation in skeletal muscle without SNS. In the present study, however, we did not identify the upstream signals of p-CREB1, which could help to predict other CREB1 phosphorylation pathways, such as cAMP-PKA or Ca2+/calmodulin (CaM)-Ca2+/calmodulin-dependent protein kinase (CaMK).

To confirm the interaction between p-CREB1 and CBP, IP experiments were performed in vitro. The interaction between p-CREB1 and CBP was increased, and indicated that cold stimulation induced the recruitment of CBP to p-CREB. In this process, the transcription and induction of CREB-targeting genes increase [15,16]. CBP is a transcriptional coactivator and an acetyltransferase that serves as a bridge for DNA-bound transcription factors (activators). It forms a basal transcription machinery through direct interactions, acts as a histone acetyltransferase to relax histone, and is an epigenetic regulator enhancing transcription [17]. Therefore, downstream responses to optimal cold stimulation may link epigenetic regulation to gene expression in muscle tissues.

Sarver et al. [18] performed a detailed investigation of the effects of local cryotherapy, using metabolome and transcriptome analyses. A single 15-min ice cup massage of the anterolateral thigh in normal human subjects had virtually no effect on muscle metabolomics or transcriptomics. Therefore, the authors suggested that acute local cryotherapy has nearly no biological effect on muscle. In the present study, however, CREB transcription increased with CREB-targeting gene upregulation. The number of cold stimulations applied differed between the present study and that of Sarver et al. [18]. Our previous study demonstrated that temperature significantly influences the extent to which cold stimulation elicits biological effects. The optimal cold temperature range was 4–17 °C [10]. Therefore, the best results of local cryotherapy of human muscle are probably obtained when cold stimulation is repeated two or three times at 4–17 °C.

Two or three 15-min cold stimulations markedly enhanced CREB1 phosphorylation, especially in C2C12 myoblasts and 3T3-L1 fibroblasts in vitro. One to three 15-min cold stimulations substantially increased CREB1 phosphorylation in TA muscle in the ex vivo and in vivo experiments. However, the extent of CREB1 phosphorylation was independent of the number of cold stimulations applied. In the ex vivo experiments, the CREB-targeting genes such as *Pgc1-α*, *Glut4*, *Creb1*, and *Cpt1b* were significantly upregulated when cold stimulations were applied only two or three times for 15 min each time. Taken together, the foregoing findings indicate that the number of cold stimulations significantly affects CREB1 activation in local cryotherapy.

In vivo experiments revealed that the mitochondrial DNA copy number, the genes associated with mitochondrial components, complexes, and biogenesis, and the CREB-targeting genes were considerably increased in response to three 15-min cold stimulations per day for 9 days. In contrast, single and short-term cold stimulations had virtually no effect on any of the aforementioned parameters. Therefore, the repetition of cold stimulation for longer than one day was also necessary to elicit CREB1 activation and mitochondrial biogenesis. However, the *Pgc-1a* gene expressions on cold stimulations group after 9 days was not changed compared with the control group. Conversely, at 1 h following the three 15-min cold stimulations, the gene expression of *Pgc-1a* was significantly increased in the treated group, compared with the control group. The samples of cold stimulations group at 9 days was harvested at 12 h from last cold stimulation. Therefore, cold induced *Pgc-1a* expression would return to baseline levels at an early stage, and it is possible that the remaining increased protein of *Pgc-1a* can induce the expressions of genes of the mitochondrial component, complex, and biogenesis. In order to reveal these unclear phenomena, further analysis by WB using antibodies against each protein is necessary. For acute, short-term local cryotherapy in vivo, only the expression of *Ndufs1*, a major protein encoded in mitochondrial complex 1, was markedly altered. Therefore, this gene could possibly become a more sensitive gene upon cold stimulations.

In a wound healing experiment, it was found that mitochondrial dysfunction induced by embelin hindered tissue repair [19]. CREB enhanced tissue repair by inducing the secretion of WNT1-inducible signaling protein 1 (WISP-1) [20]. Activated CREB also enhanced cell migration [21]. Excessive reactive oxygen species (ROS) production or impaired ROS detoxication results in oxidative damage, which impedes chronic wound healing [22]. In the present study, optimal cold stimulation activated CREB1 and mitochondrial biogenesis, and upregulated antioxidant genes. Therefore, optimal cryotherapy may stimulate tissue repair following sprains, pulled muscle, and bruises.

It was reported that cryotherapy for tissue injury [6] and cold stimulation for cellular repair [23] applied for >24 h hinders tissue and cell recovery, respectively. ROS produced in response to DNA damage and cell death increased when the temperature of the cell culture was lowered to 25 °C, maintained there for 5 days, then was raised to 37 °C [24]. In the present study, however, 15-min cold stimulations repeated several times did not induce cell damage in vitro or tissue degradation in vivo. Moreover, the levels of TBARS were unaltered, which indicate chronic oxidative stress in muscle. Therefore, both short-term and longer-term cold stimulations as intermittent would be safe and effective in vivo and in vitro.

There are some limitations to this study. We could not measure to what temperature the muscle tissue was cooled in ex vivo and in vivo experiments. It could be important to measure and regulate the exact temperature of the intra-muscular region to reveal accurate molecular mechanisms of local cryotherapy under the condition setting with constant cooled muscle temperature. Moreover, analysis of the intra-muscular temperature using a delicate thermometer is desirable to confirm whether the activation of CREB seen in this study agrees with the three models of in vitro, ex vivo, and in vivo experiments in this study. However, the observations in the in vitro, ex vivo, and in vivo experiments of increasing levels of induced CREB phosphorylation upon repeated cold stimulations support the view that it is possible to interpret the results comprehensively, integrating the findings from the experimental designs.

We used only cell lines and animals. Therefore, the knowledge gained here cannot be directly applied to human subjects. To clarify the true effect of local cryotherapy on human muscles, a randomized controlled trial involving cryotherapy as a treatment for muscle trauma or fatigue is needed. If a randomized controlled trial is performed on human subjects, the cold stimulation temperature and applied number of stimulations may be important factors for eliciting an effect from local cryotherapy. This is because we presently discovered that the expression of CREB-targeting genes and transcriptional activation of CREB were dependent on repeated numbers of cold stimulation both in vitro and in vivo. Moreover, in our previous study, we discovered that cold stimulation increased mitochondrial activities, dependent on cold temperature [10]. In addition, it could be hypothesized that, because the temperature and stimulation numbers differed among previous in vivo studies, consistent results have not been obtained [2,3,4,5,6,7,8]. In order to solve these problems, the consistent management of cooling temperatures and application methods may be essential for a human study of local cryotherapy.

**Figure 8:**
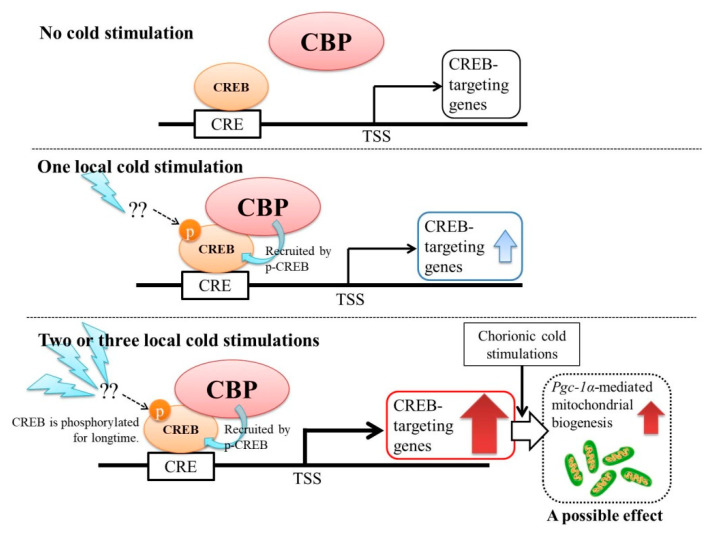
This model proposes the molecular mechanisms of local cryotherapy. CREB: cAMP response element-binding protein; CBP: CREB-binding protein; CRE: cAMP-responsive element; TSS: Transcription start site.

Taken together, the present study demonstrates that optimal local cold stimulations activate CREB transcription and increase its downstream reactions. It is possible that local cold stimulations partially activate mitochondrial biogenesis in local muscle without SNS involvement. In addition, the magnitudes of these effects were commensurate with the frequency and duration of the cold stimulations. Our model is presented in the form of Figure 8 (also as graphical abstract). The discoveries reported in the present study could accelerate the refinement and practical application of local cryotherapy.

## 4. Materials and Methods

### 4.1. In Vitro Experiments

#### 4.1.1. Cell Lines

Four cell lines that contribute to muscle tissue composition, and one cell line used to analyze protein-protein interaction and transcriptional activities by transfection of nucleic acids, were used in the present study. These included mouse myoblasts (C2C12 cells: RCB0987, Riken BRC Cell Bank, Tsukuba, Ibaraki, Japan) mouse embryonic fibroblasts (3T3-L1 cells: JCRB9014, JCRB Cell Bank, Ibaraki, Osaka, Japan; original developers: Green et al.), human embryonic fibroblasts (HF cells: JCRB 1006.7, JCRB Cell Bank; original developers: Kouchi and Namba), rat skeletal muscle myoblasts (L6 cells: JCRB 9081, JCRB Cell Bank; original developer: Yaffe), and human embryonic kidney cells (HEK 293 cells; Thermo Fisher Scientific, Waltham, MA, USA). All cell lines were cultured in Dulbecco’s modified Eagle’s medium (DMEM; Thermo Fisher Scientific) containing 10% FetalClon III serum (GE Healthcare, Chicago, IL, USA), penicillin, and streptomycin, and incubated at 37 °C, 100% relative humidity, and 5% CO_2_.

#### 4.1.2. Cold Stimulation of Cells

In our previous in vitro study [10], since stimulations with cold water set at 4 °C most affected mitochondrial activity and biogenesis, the same condition was performed for each cell line in this study. Presently, the number of repetitions and the timing of evaluations were changed. The cold stimulation of each number of repetitions was induced by decreasing medium temperature from 14 to 9 °C [10]. In brief, the C2C12, 3T3-L, HF, and L6 lines were seeded at a density of 2 × 10^5^ cells in six-well culture plates containing 2 mL medium per well (*n* = 2 per group) and incubated at 37 °C. After 24 h, the culture plates were wrapped in polyethylene bags and placed for 15 min in a shaded container holding water cooled to 4 °C. This chilling process was performed once, twice, or three times, with 15-min intervals between coolings. The control cells were placed in an incubator at 37 °C. During the intervals, the cells were maintained in the incubator at 37 °C. After each cold stimulation, and at each time point, cells were harvested and subjected to western blotting, to detect inducing CREB phosphorylation. An overview of these experimental procedures is presented in Supplemental Figure S1a.

#### 4.1.3. Western Blotting (WB)

Cells subjected to cold stimulations were dissolved in lysis buffer (50 mM Tris-HCl (pH 7.4), 150 mM NaCl, 1% NP40, 1 mM EDTA, 10 mM NaF, and 2 mM Na_3_VO_4_) with protease inhibitor cocktail (Nakalai Tesque, Nakagyo, Kyoto, Japan). The cell lysates were centrifuged at 12,000× *g* for 15 min at 4 °C, and total protein in the supernatants was measured with a bicinchoninic acid (BCA) protein assay kit (TaKaRa Bio, Kusatsu, Shiga, Japan). The protein concentrations were adjusted to 2 mg/mL with SDS-PAGE buffer and the samples were heated to 95 °C for 5 min. Ten microgram protein samples were subjected to 10% SDS-PAGE at 140 V for 80 min. The gel-bound proteins were applied to polyvinylidene fluoride (PVDF) membranes by the wet transfer method, and run overnight at 40 V. Target protein bands ware detected using the primary antibodies anti-phosphorylated-CREB (p-CREB) (#9189; Cell Signaling Technology, Danvers, MA, USA), anti-CREB (#9189; Cell Signaling Technology), and anti-glyceraldehyde 3-phosphate dehydrogenase (GAPDH) (sc-32233; Santa Cruz Biotechnology, Dallas, TX, USA) and the secondary horseradish peroxidase (HRP)-linked antibodies anti-mouse IgG (#7076; Cell Signaling Technology) and anti-rabbit IgG (#7074; Cell Signaling Technology) in a luminescence detector. All WB raw data are provided as Supplemental Data 1.

#### 4.1.4. Cell Damage Assessment

With regard to Section 4.1.2, the C2C12, 3T3-L, HF, and L6 cells were seeded and the cold stimulations were performed. Twenty-four hours after the final cold stimulation, cell morphology was examined and a trypan blue assay was performed using a hemocytometer (*n* = 4 per group) to evaluate cell damages by cold stimulations, as previously described [10].

#### 4.1.5. Immunoprecipitation (IP)

Since the HEK 293 cells have high transfection efficiency, this cell line was used in this experiment. To analyze the interaction between p-CREB and CREB-binding protein (CBP; a CREB coactivator), HEK 293 cells were seeded at a density of 2 × 10^5^ in a six-well plate containing 2 mL culture medium per cell and incubated at 37 °C. After 24 h, the cells were transfected with 6 μg of pcDNA3β-FLAG-CBP-HA plasmids (source: Tso-Pang Yao; Addgene plasmid No. 32908; http://n2t.net/addgene:32908; RRID:Addgene_32908), using Polyethylenimine Max (Polysciences, Warrington, PA, USA), and the existing medium was replaced with DMEM, containing 1% FetalClon III serum and antibiotics (serum reduction medium) to enhance transfection efficacy. After 12 h, the serum reduction medium was replaced with normal medium, and the cells were incubated at 37 °C for 24 h. The cells were then subjected to cold stimulations, as previously described. Ten minutes after the final cold stimulations, the cells were harvested and lysed, and their protein concentrations were measured, as previously described. Five hundred micrograms of total protein were mixed with 25 μL of Pierce Anti-HA Magnetic Beads (Thermo Fisher Scientific). The samples were incubated with agitation at 4 °C overnight, and then rinsed with a buffer consisting of 50 mM Tris-HCl (pH 7.4), 150 mM NaCl, 0.5% NP40, and 1 mM EDTA. The beads were boiled in SDS sample buffer to separate the antibody and antigen. In an independent experiment, a positive control sample to enhance the recruitment of CBP to p-CREB was made by applying 50 μM forskolin (FSK; Tokyo Chemical Industries, Chuo, Tokyo, Japan) for 30 min. Interactions between p-CREB and CBP were analyzed by WB, as previously described. Anti-CBP (sc-7300; Santa Cruz Biotechnology) and anti-human influenza hemagglutinin (HA) tag antibody (041-21881; Wako Pure Chemical Industries, Osaka, Osaka, Japan) were also used in the WB analysis.

#### 4.1.6. Reporter Assay Using cAMP Response Elements

HEK 293 cells were also used in this experiment for the same reason described above. To analyze the transcriptional activity of CREB in response to the cold stimulations, a reporter assay was performed. The repeat sequences of the cAMP response elements (CRE; 5′-TGACGTCA-3′) were cloned to pGL3-Basic Vector (Promega, Madison, WI, USA). HEK 293 cells were seeded at a density of 0.25 × 10^5^ in a 24-well plate containing 500 μL culture medium per well (*n* = 4 per group) and incubated at 37 °C. After 24 h, the cells were transfected with CRE-pGL3-Basic vector plasmids (750 μg) using Polyethylenimine Max. The existing medium was replaced by serum reduction medium consisting of DMEM containing 1% FetalClon III and antibiotics to enhance transfection efficacy. After 12 h, the serum reduction medium was replaced with normal medium, and the cells were incubated at 37 °C for 24 h. The cells were then subjected to cold stimulations, as previously described. Ten minutes after the final cold stimulation, a reporter assay was performed using a dual-luciferase reporter assay system (Promega), according to the manufacturer’s instructions. Total protein content was also measured with a Pierce Coomassie Plus (Bradford) assay kit (Thermo Fisher Scientific), to normalize the luminescence intensity.

### 4.2. Ex Vivo and In Vivo Experiments

#### 4.2.1. Animals

All animal experiments were approved by the Animal Care Committee, University of Tsukuba (No. 18-071). Forty-eight male CBA/J mouse (4 weeks; average weight 14.7 ± 1.2 g) and 26 ICR mice (9–11 weeks; average weight 40.8 ± 1.8 g) were used in this study. All animals were maintained in a temperature-controlled environment with a 12-h light/dark cycle, and given free access to standard laboratory food and water.

#### 4.2.2. Cold Stimulations for Skeletal Muscle Ex Vivo

An overview of the experimental procedures is shown in Supplemental Figure S1b. Both TA muscles were harvested from 24 euthanized CBA/J mice (*n* = 4 per group), immersed in a 2-mL micro tube, containing 1 mL of DMEM with 10 mM HEPES (pH 7.4), 10% FetalClon III, and antibiotics, and pre-incubated at 37 °C for 20 min. The micro tubes containing the right TA muscles were immersed for 15 min in a shaded container, containing water cooled to 4 °C. This step was performed once, twice, or thrice. There were 15-min intervals between each cooling during which the muscle tissue was kept in an incubator at 37 °C. The left TA muscles were incubated at 37 °C as a control. Ten minutes after the final cold stimulation, the TA muscles were homogenized in lysis buffer containing a protease inhibitor cocktail. The protein concentrations were measured and 10 μg protein was subjected to WB to detect p-CREB, CREB, and CBP, as previously described. In an independent assay, Sepasol-RNA I Super G (Nakalai Tesque) was used, according to the manufacturer’s instructions, to extract total RNA from each TA muscle sample 1 h after the final cold stimulations (*n* = 4 per group). Reverse transcription and cDNA generation were performed with 500 ng total RNA and PrimeScrip RT Master Mix (Takara Bio). The cDNA was diluted 10× with nuclease-free water. Quantitative PCR (qPCR) was performed with a KAPA SYBR Fast qPCR kit (Nippon Genetics, Bunkyo, Tokyo, Japan), to evaluate the expression levels of the genes targeted by CREB. The 18 s ribosomal RNA expression was also measured and gene expressions were normalized by the 2^−ΔΔCt^ method. Primer sequences and gene full names are presented in Supplemental Table S1. CREB-targeting genes involved in mitochondrial biogenesis and metabolic regulators, such as peroxisome proliferator-activated receptor gamma coactivator 1-alpha (*Pgc-1α*), cAMP-responsive element binding protein 1 (*Creb1*), glucose transporter type 4 (*Glut4*), and carnitine palmitoyltransferase 1b (*Cpt1b*), were selected and measured on the basis of earlier studies [16,25,26,27].

#### 4.2.3. Cold Stimulations for In Vivo Experiments

We performed two in vivo experiments as described below. An overview of the experimental procedures is shown in Supplemental Figure S1c.

##### Cold Exposure of Legs

ICR mice were placed in an in-house-designed holding device and anesthetized by isoflurane inhalation. The right limb of each mouse was immersed to the knee for 15 min, in water cooled to 4 °C. This treatment was repeated twice, with 15-min intervals between cold stimulations, during which the mice were awakened and kept in a breeding cage. The right limbs of the control mice were placed in empty containers under the isoflurane inhalation in the holding device.

##### Cold Exposure Experiments to Confirm CREB Phosphorylation and Induced Expressions of CREB-Targeting Genes

To confirm whether CREB phosphorylation and expressions of CREB-targeting genes are induced, the treatments were applied once. Ten minutes after the final cold stimulation, the TA muscles were harvested (*n* = 4 per group) and kept in liquid nitrogen until further sample preparations. TA muscles were homogenized in lysis buffer containing a protease inhibitor cocktail. The protein concentrations were measured and 10 μg protein was subjected to WB to detect p-CREB, CREB, and GAPDH, as previously described. In an independent assay, Sepasol-RNA I Super G was used, according to the manufacturer’s instructions, to extract total RNA from tibialis anterior (TA), extensor digitorum longus (EDL), lateral head of gastrocnemius (LG), and medial head of gastrocnemius (MG) muscle sample (*n* = 12 per group). One hour after the final cold stimulations, reverse transcription, cDNA generation, and qPCR were performed to evaluate the expression level of CREB-targeting genes, as previously described.

##### Acute and Chronic Cold Exposure for Legs Followed Each Analysis

For the acute cold exposure experiment, the treatments were applied for 1 day. For the chronic cold stimulation experiment, the treatments were applied once daily for 9 consecutive days.

Twelve hours after the final cold stimulation, the animals were euthanized by cervical dislocation under anesthesia induced using inhaled isoflurane, and the limb muscles, TA, EDL, LG, and MG were individually harvested (*n* = 12 per group). Total RNA was extracted and qPCR assay was performed, as previously described. Based on earlier studies [25,28,29,30] *Pgc1a*-regulated, mitochondrial complex, and component genes were selected and measured in this experiment, because the in vitro and ex vivo experiments in this study showed that CREB phosphorylation and gene expressions of CREB-targeting gene including *Pgc-1a* were induced upon cold stimulations. These primer sequences and gene full names are presented in Supplemental Table S1.

Biceps femoris and gracilis on the surface of the lateral and medial heads of the gastrocnemius muscle were also harvested (*n* = 6 per group), and their total DNA was extracted with phenol/chloroform/isoamyl alcohol (25:24:1; Nakalai Tesque), according to the manufacturer’s instructions. The total DNA of each sample was adjusted to 10 ng/μL. Using the total DNA, the mitochondrial DNA (mtDNA) copy numbers were measured by qPCR assay, as previously described. The primer sequences for mtDNA and nuclear DNA (nDNA) are shown in Supplemental Table S1. The mtDNA abundance was calculated using the 2^−ΔΔCt^ method to nDNA, and relative values were also calculated. The right feet and total muscle protein were harvested to assess any adverse effects associated with the cold stimulations.

The mouse feet were sectioned at a thickness of 3 μm, embedded in paraffin, stained with hematoxylin and eosin (H&E), and their muscle, bone, and skin components were examined under a model BZ-X710 optical microscope (Keyence, Osaka, Osaka, Japan) to assess adverse effects, such as cold induced inflammation.

The total protein of 100 μg each muscle tissue (TA, ELD, LG, and MG; *n* = 12 per group) was subjected to a thiobarbituric acid-reactive substances (TBARS) assay, to measure lipid peroxidation referring oxidative stress by reactive oxygen species (ROS). TBARS were measured according to the method of Kikugawa et al. (2003), with certain modifications, as follows. One hundred microliters of total protein (1 mg/mL) was added to a screw-cap tube containing a mixture of 325 μL thiobarbituric acid (TBA) with 2 mL of 5.2% (*w*/*v*) sodium dodecyl sulfate (SDS) in distilled water (DW), 500 μL of 0.8% (*w*/*v*) butylated hydroxytoluene (BHT) in glacial acetic acid, 15 mL of 0.8% (*w*/*v*) thiobarbituric acid (TBA) in DW, and 17 mL of DW alone. Then 75 μL acetate buffer (pH 3.5) was added to the tube, to make up a final volume of 500 μL. The tube was stored at 4 °C for 60 min, then heated at 95 °C for 60 min. Then, 500 μL of 15:1 *v*/*v* 1-butanol and pyridine was added to the chilled tube, and the mixture was centrifuged at 3000 rpm for 10 min at 4 °C. Fluorescence of the supernatant was measured at 540 nm excitation and 590 nm emission. The TBARS concentrations were interpolated from a regression equation plotted with a 1,1,3,3-tetraethoxypropane standard (100 to 0.4 nmol/mL)

### 4.3. Statistical Analyses

Data are presented as means ±SD. Distribution normality was verified with the Shapiro-Wilk test using GraphPad Prism v. 7.04 (GraphPad Software, San Diego, CA, USA). Normally distributed data of four groups with two factors were subjected to two-way ANOVA, followed by the Bonferroni correction as a post-hoc test, using the same software. Normally distributed data of two groups with one factor were subjected to Welch’s *t*-test by Excel 2010 (Microsoft, Redmond, WA, USA). *p* < 0.05 was considered statistically significant.

## Supplementary Materials

Supplementary materials can be found at https://www.mdpi.com/1422-0067/21/13/4588/s1.
