# Supplementary material for: Influence of Intermittent Cold Stimulations on CREB and Its Targeting Genes in Muscle: Investigations into Molecular Mechanisms of Local Cryotherapy
Source: Int J Mol Sci. 2020 Jun 28;21(13):4588. doi: 10.3390/ijms21134588 (PMC7370117; doi:10.3390/ijms21134588)
Supplement: Supplementary file 1 [file ijms-21-04588-s001.zip › Supplemental figs, table and data1/supplemental data 1_revise-2/Explanatory data for WB.pptx]

## Slide 1
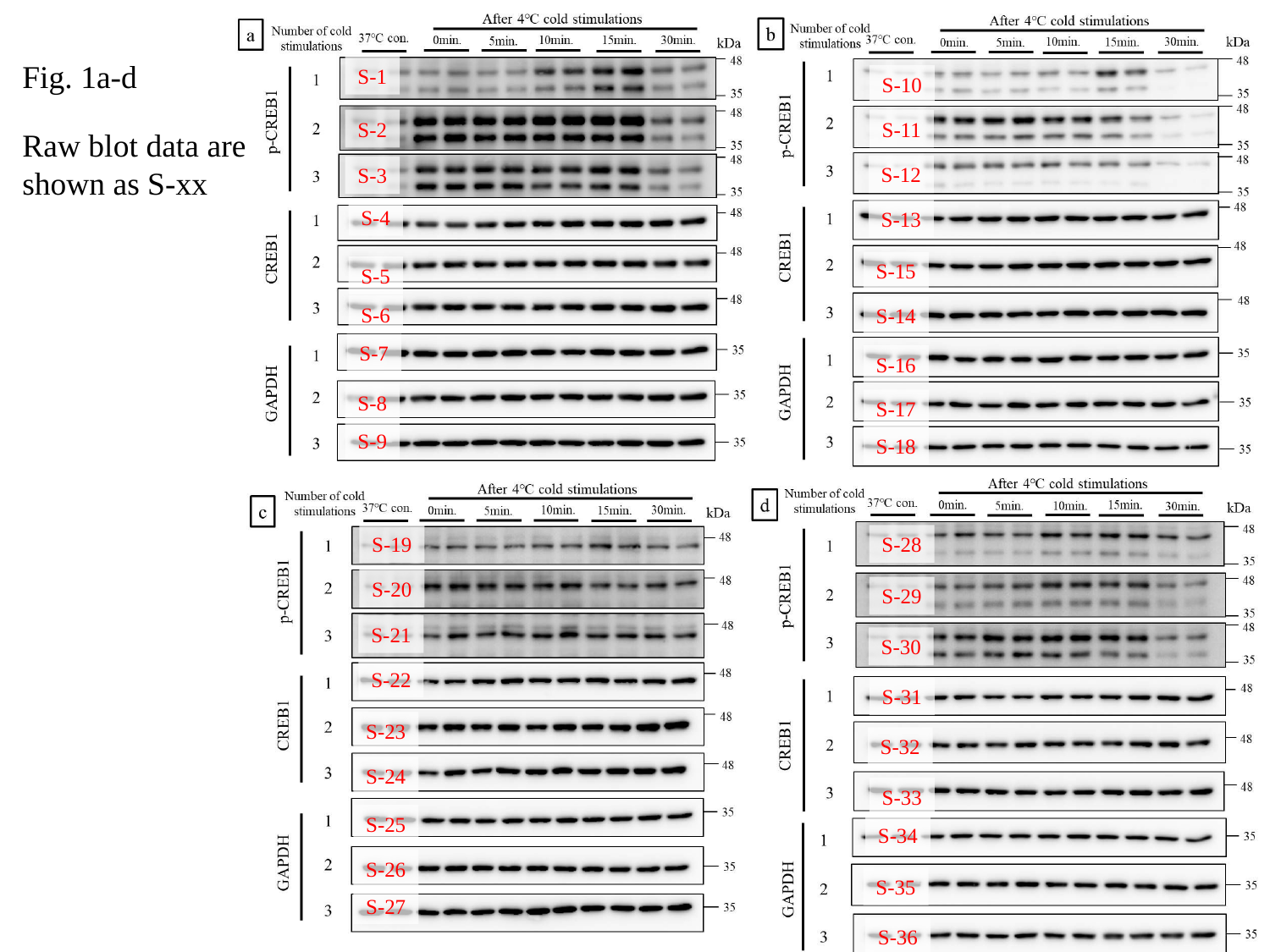

Fig. 1a-d
S-1
S-10
S-2
S-11
Raw blot data are shown as S-xx
S-12
S-3
S-4
S-13
S-15
S-5
S-6
S-14
S-7
S-16
S-8
S-17
S-9
S-18
S-28
S-19
S-20
S-29
S-21
S-30
S-22
S-31
S-23
S-32
S-24
S-33
S-25
S-34
S-26
S-35
S-27
S-36

## Slide 2
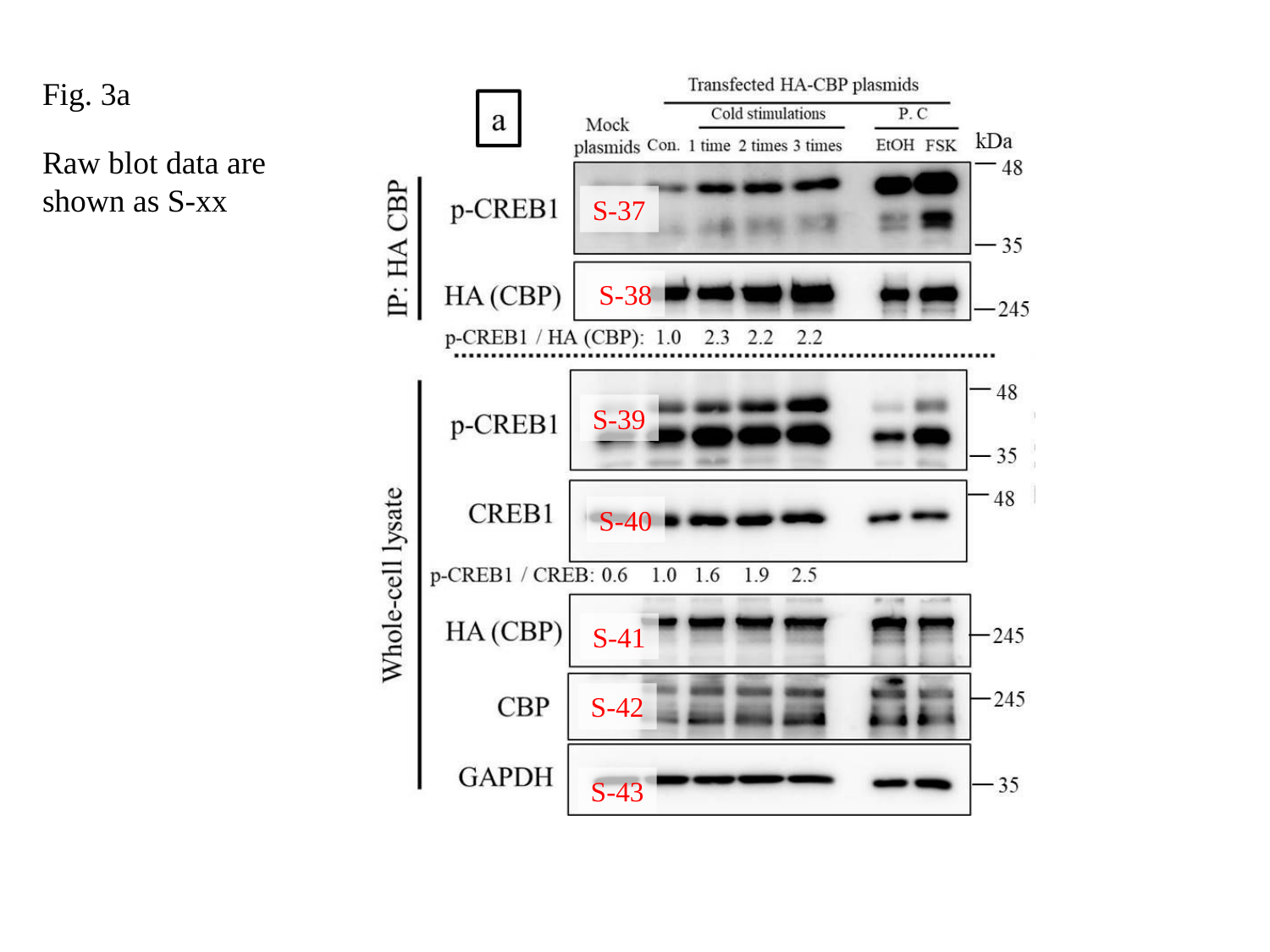

Fig. 3a
Raw blot data are shown as S-xx
S-37
S-38
S-39
S-40
S-41
S-42
S-43

## Slide 3
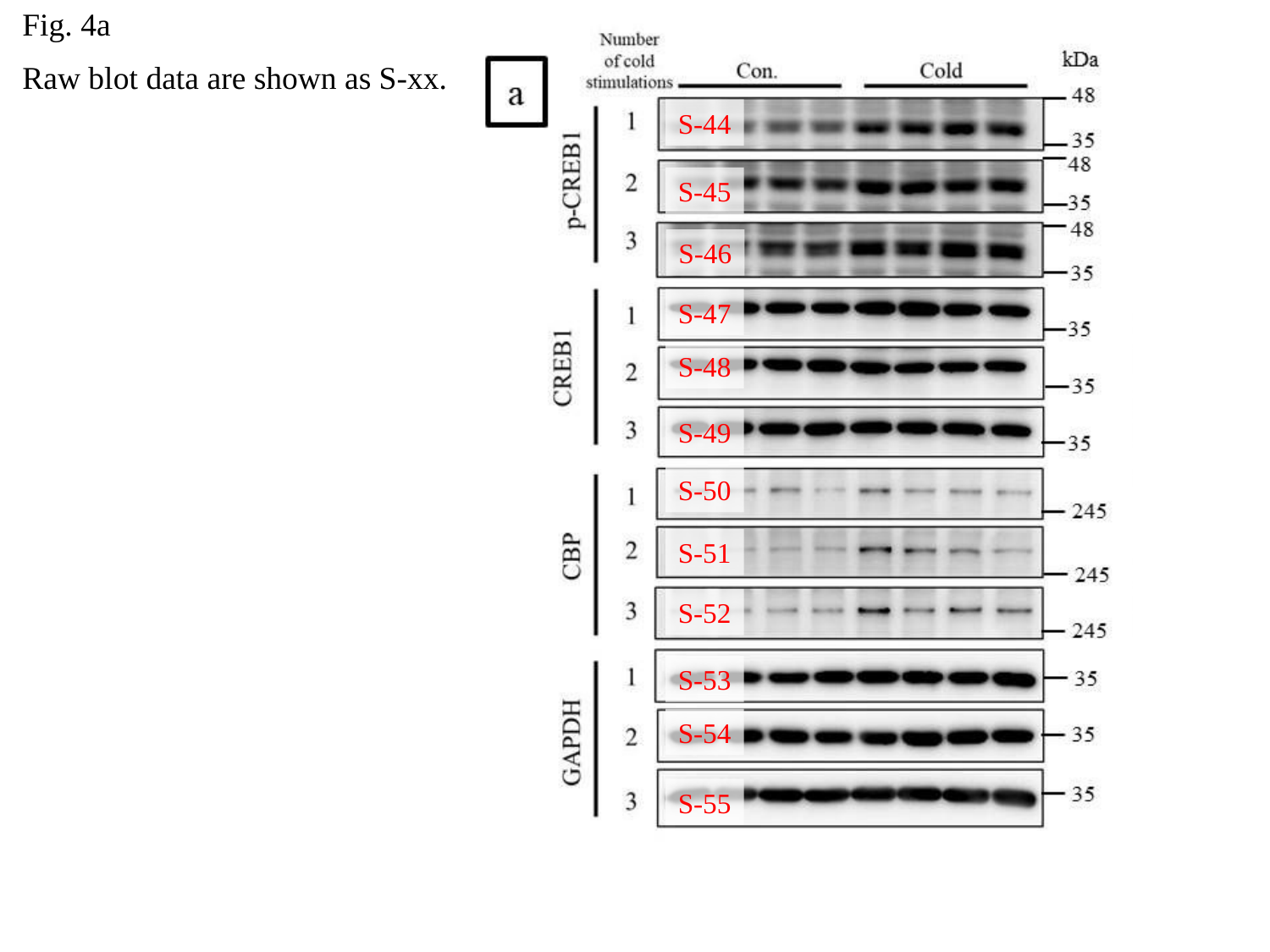

Fig. 4a
Raw blot data are shown as S-xx.
S-44
S-45
S-46
S-47
S-48
S-49
S-50
S-51
S-52
S-53
S-54
S-55

## Slide 4
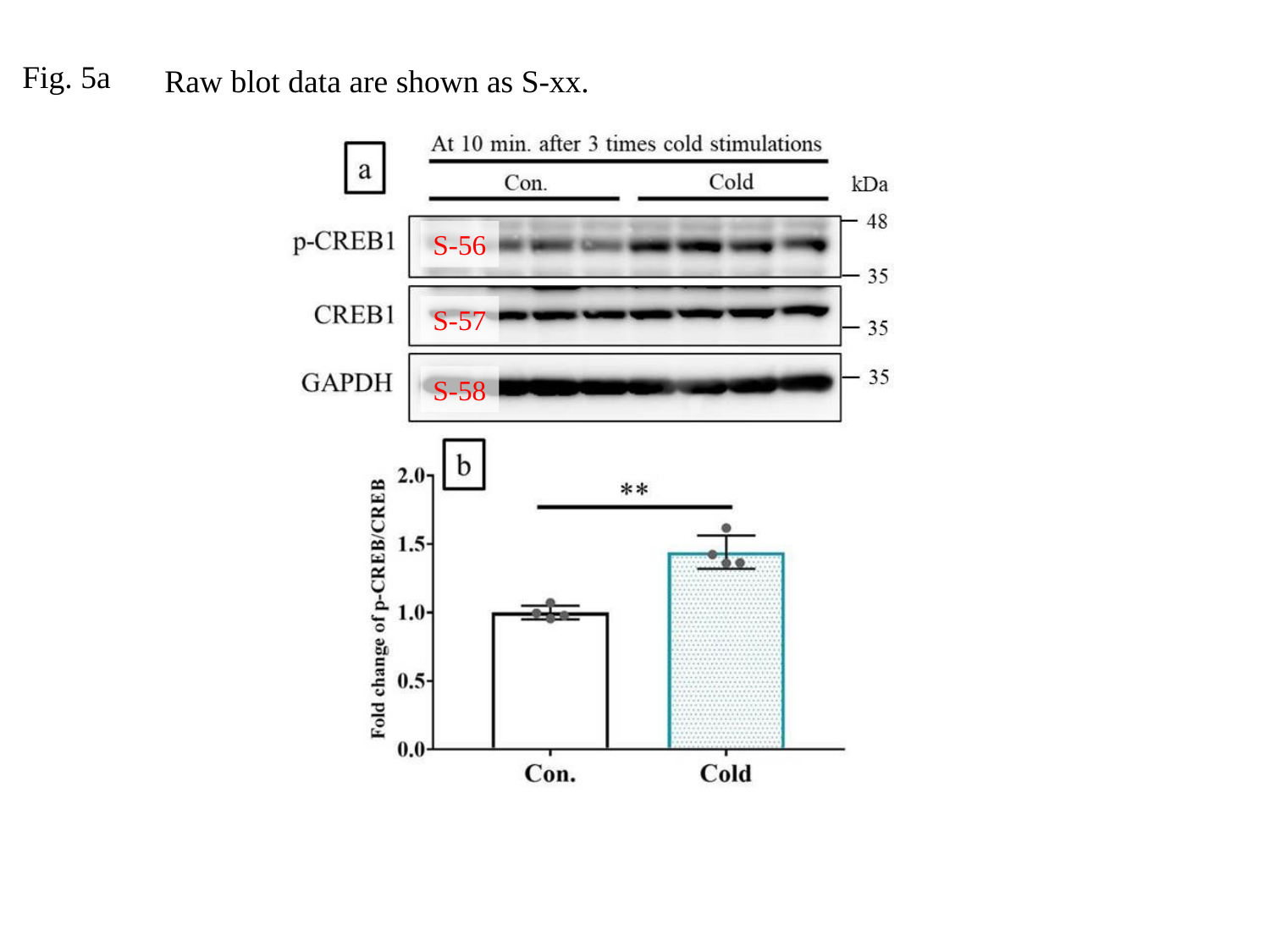

Fig. 5a
Raw blot data are shown as S-xx.
S-56
S-57
S-58
