# Supplementary material for: Influence of Intermittent Cold Stimulations on CREB and Its Targeting Genes in Muscle: Investigations into Molecular Mechanisms of Local Cryotherapy
Source: Int J Mol Sci. 2020 Jun 28;21(13):4588. doi: 10.3390/ijms21134588 (PMC7370117; doi:10.3390/ijms21134588)
Supplement: Supplementary file 1 [file ijms-21-04588-s001.zip › Supplemental figs, table and data1/Supplemental Figs_revise-2.pdf]

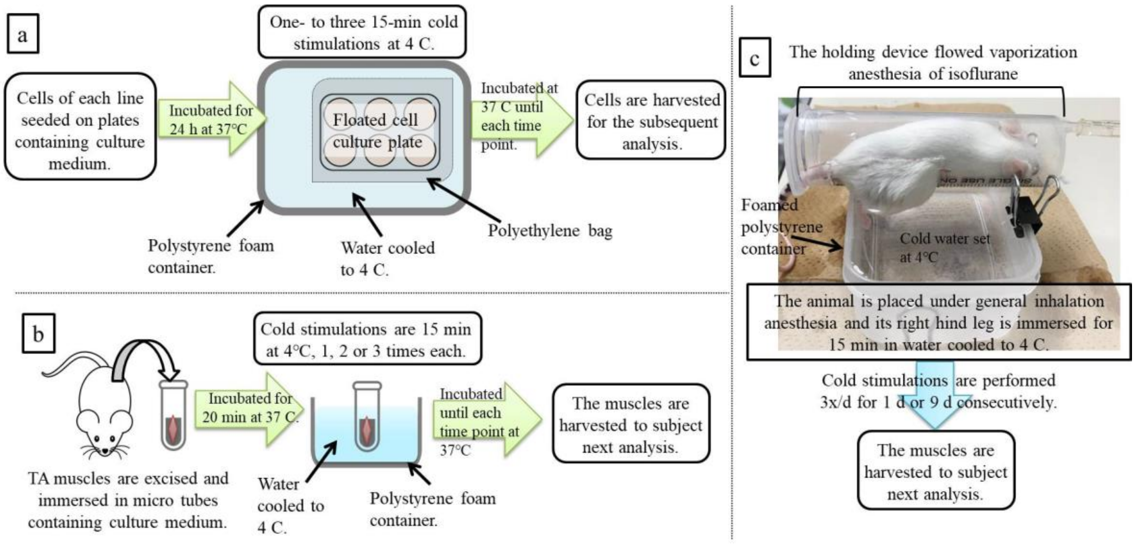

Supplemental Fig. 1. Overview of cold stimulation methods.

a: In vitro experiment; b: ex vivo experiment; c: in vivo experiment.

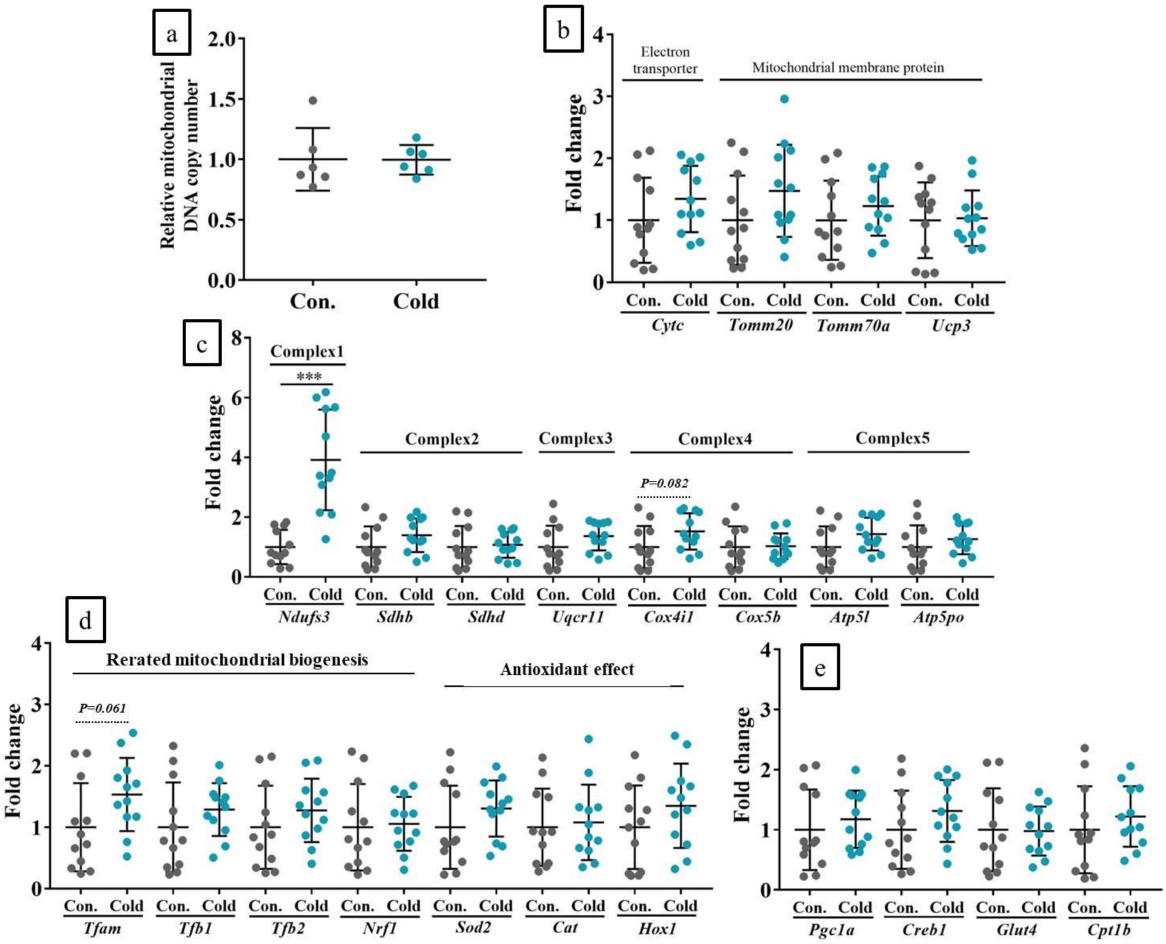

Supplemental Fig. 2. Acute cold stimulation had almost no effect on mitochondrial biogenesis or other factors in vivo.

a: Mitochondrial DNA copy number; b: mitochondrial components genes, c: mitochondrial complex genes, d: *Pgc1-α* regulated genes; e: CREB-targeting genes in acute cold stimulation in vivo. Con: control (no cold stimulation); Cold: 15-min cold stimulation. N=12 per group. ***P < 0.001 according to Welch's t-test.
