# Supplementary figures and images for: Influence of Intermittent Cold Stimulations on CREB and Its Targeting Genes in Muscle: Investigations into Molecular Mechanisms of Local Cryotherapy
Source: Int J Mol Sci. 2020 Jun 28;21(13):4588. doi: 10.3390/ijms21134588 (PMC7370117; doi:10.3390/ijms21134588)

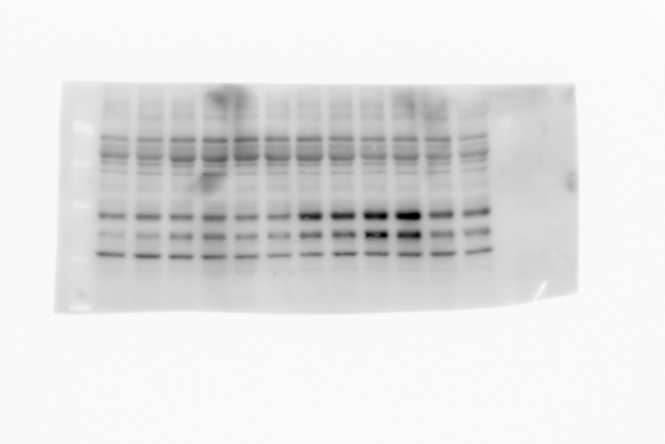

Supplement: Supplementary file 1 [file ijms-21-04588-s001.zip › Supplemental figs, table and data1/supplemental data 1_revise-2/S-1.tif]

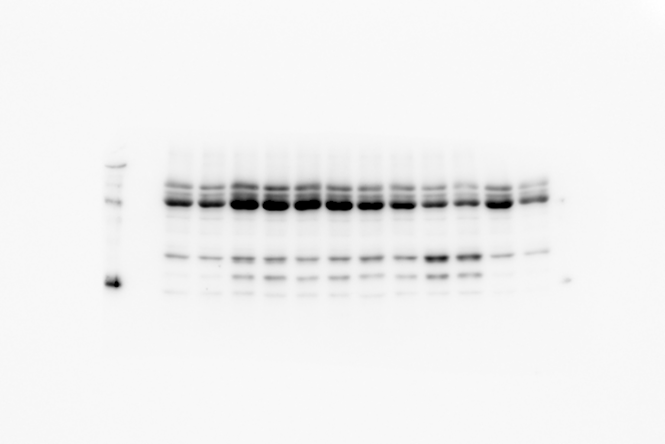

Supplement: Supplementary file 1 [file ijms-21-04588-s001.zip › Supplemental figs, table and data1/supplemental data 1_revise-2/S-10.tif]

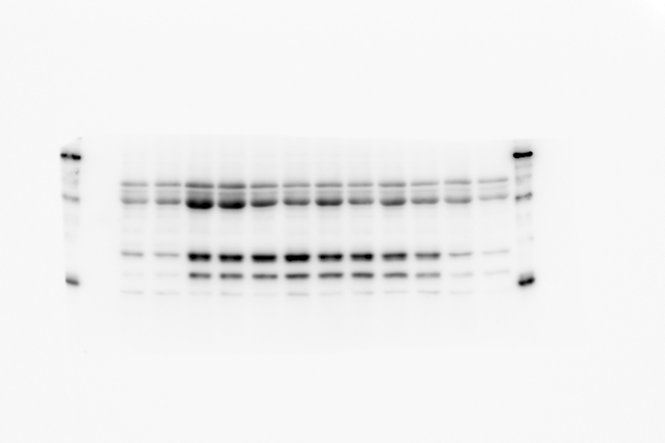

Supplement: Supplementary file 1 [file ijms-21-04588-s001.zip › Supplemental figs, table and data1/supplemental data 1_revise-2/S-11.tif]

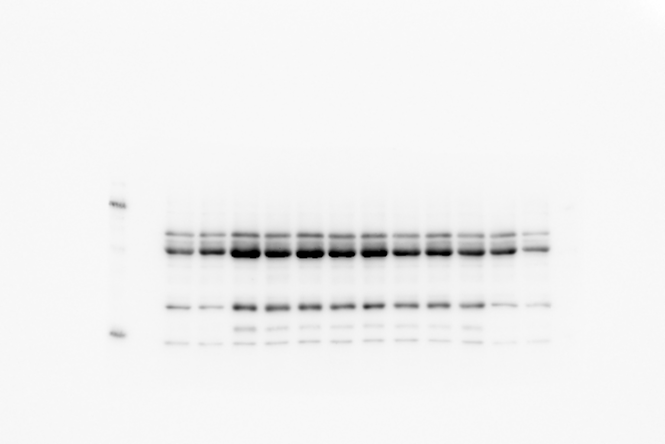

Supplement: Supplementary file 1 [file ijms-21-04588-s001.zip › Supplemental figs, table and data1/supplemental data 1_revise-2/S-12.tif]

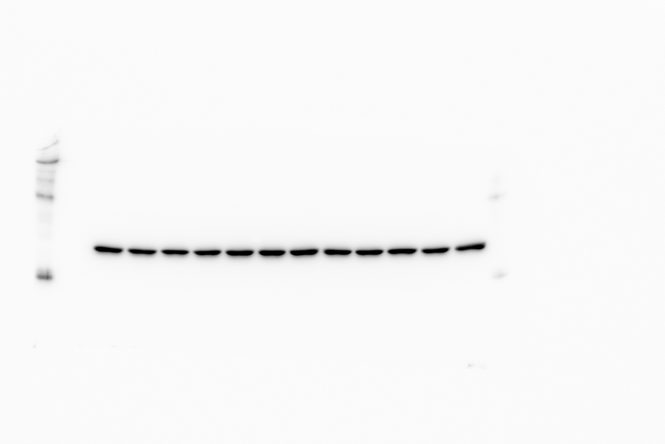

Supplement: Supplementary file 1 [file ijms-21-04588-s001.zip › Supplemental figs, table and data1/supplemental data 1_revise-2/S-13.tif]

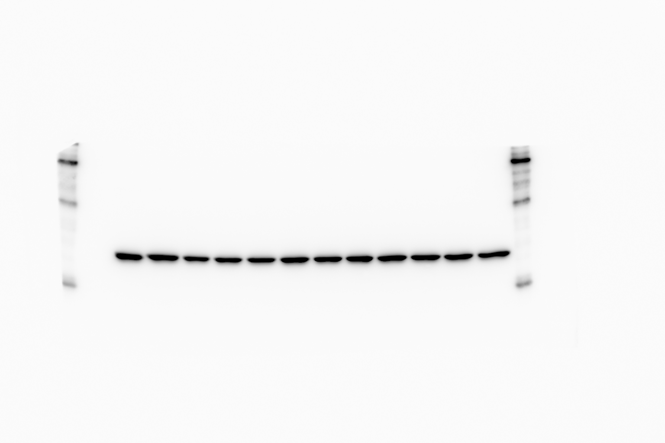

Supplement: Supplementary file 1 [file ijms-21-04588-s001.zip › Supplemental figs, table and data1/supplemental data 1_revise-2/S-14.tif]

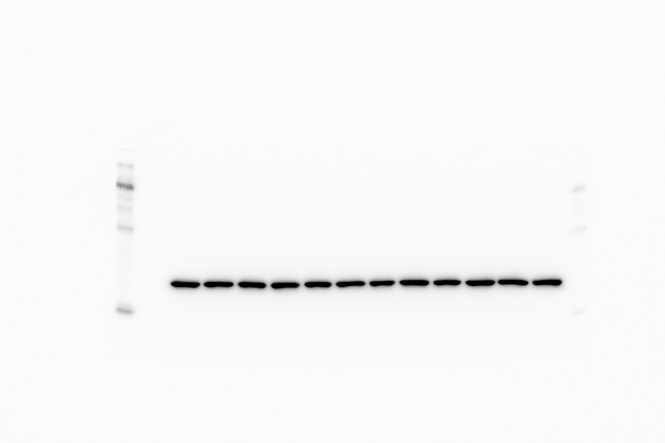

Supplement: Supplementary file 1 [file ijms-21-04588-s001.zip › Supplemental figs, table and data1/supplemental data 1_revise-2/S-15.tif]

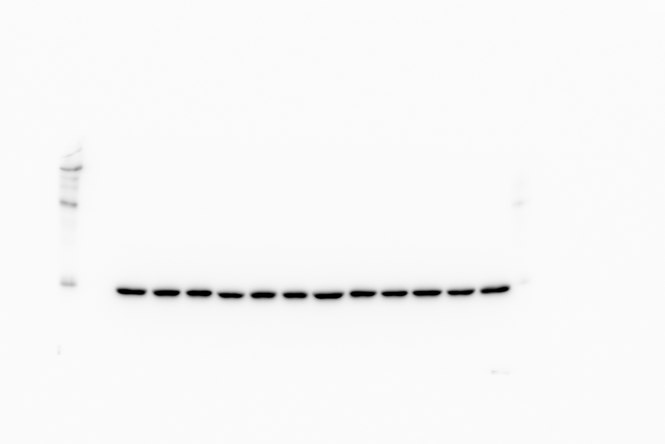

Supplement: Supplementary file 1 [file ijms-21-04588-s001.zip › Supplemental figs, table and data1/supplemental data 1_revise-2/S-16.tif]

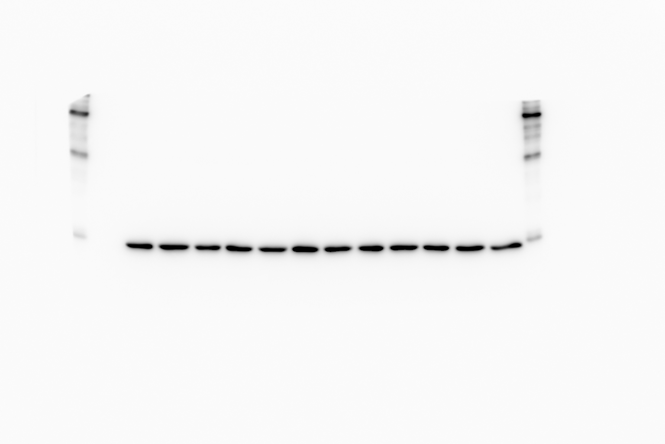

Supplement: Supplementary file 1 [file ijms-21-04588-s001.zip › Supplemental figs, table and data1/supplemental data 1_revise-2/S-17.tif]

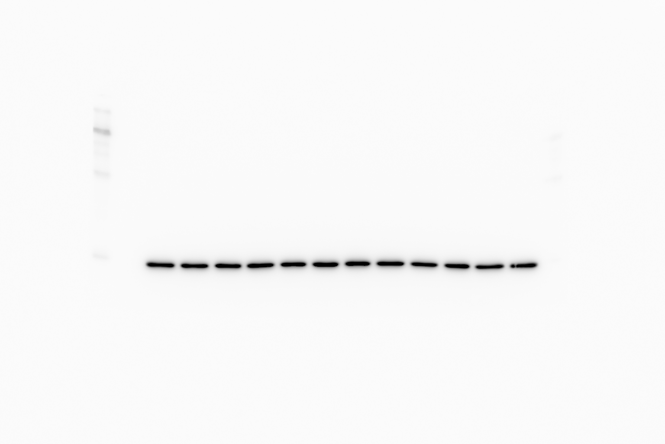

Supplement: Supplementary file 1 [file ijms-21-04588-s001.zip › Supplemental figs, table and data1/supplemental data 1_revise-2/S-18.tif]

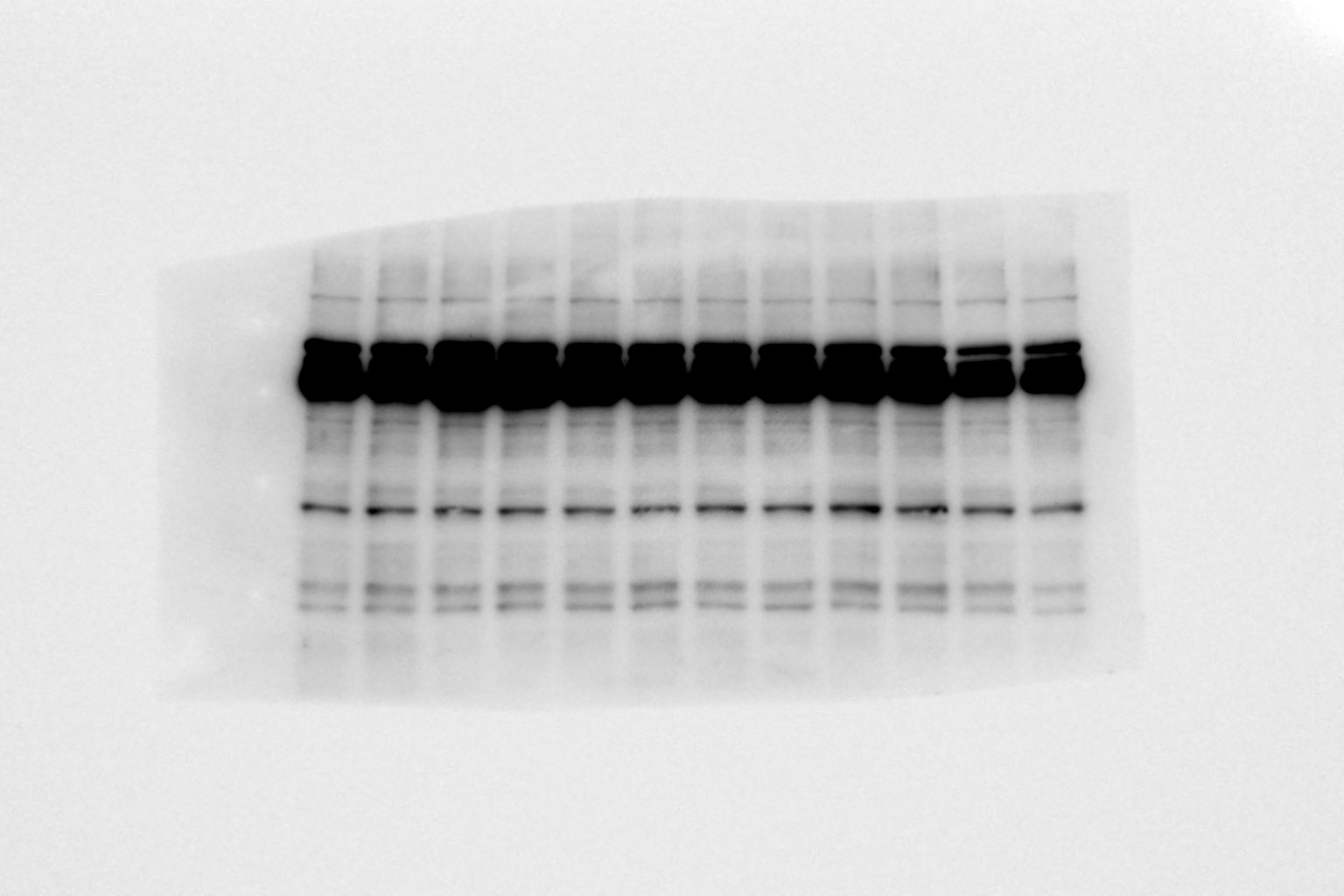

Supplement: Supplementary file 1 [file ijms-21-04588-s001.zip › Supplemental figs, table and data1/supplemental data 1_revise-2/S-19.tif]

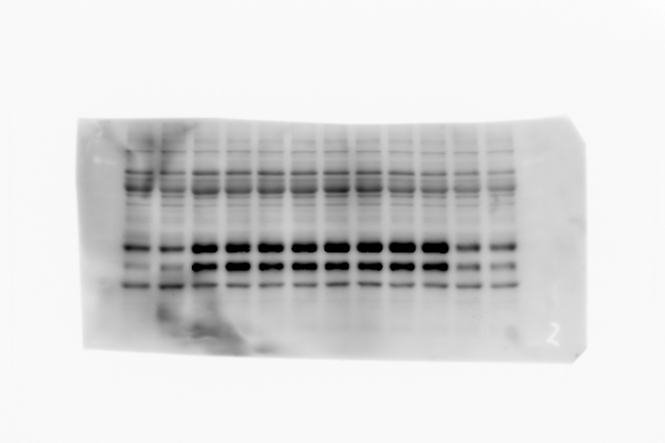

Supplement: Supplementary file 1 [file ijms-21-04588-s001.zip › Supplemental figs, table and data1/supplemental data 1_revise-2/s-2.tif]

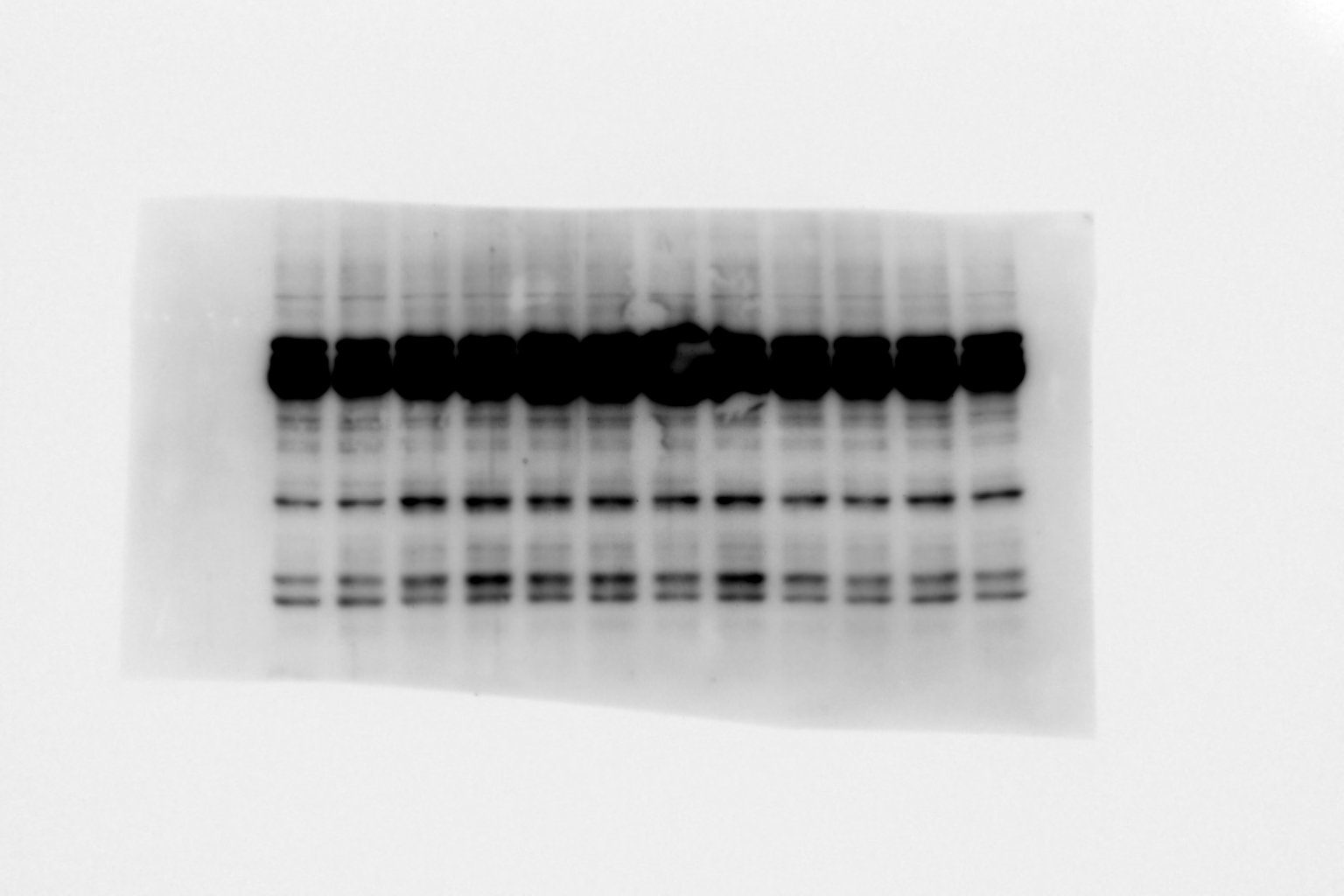

Supplement: Supplementary file 1 [file ijms-21-04588-s001.zip › Supplemental figs, table and data1/supplemental data 1_revise-2/S-20.tif]

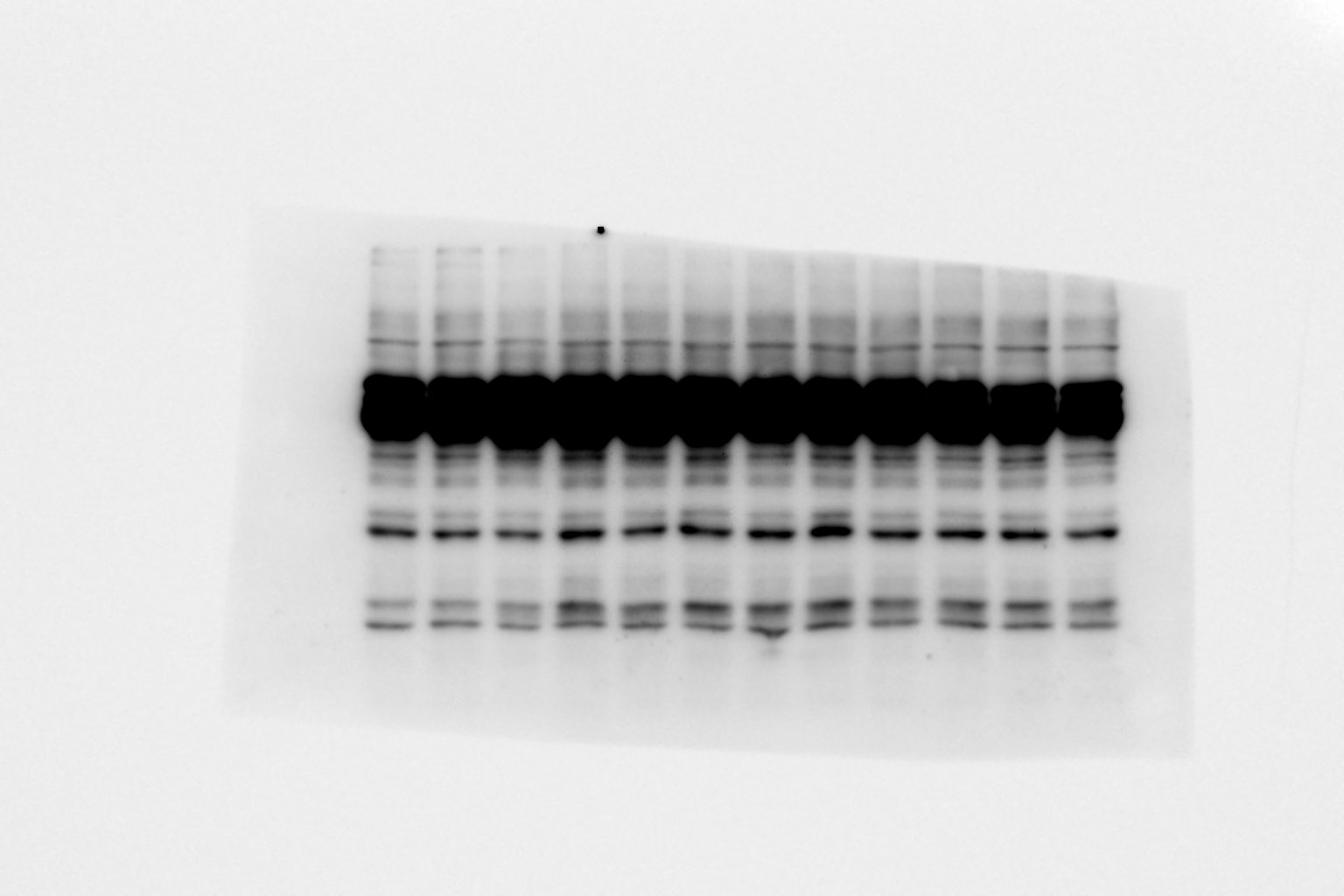

Supplement: Supplementary file 1 [file ijms-21-04588-s001.zip › Supplemental figs, table and data1/supplemental data 1_revise-2/S-21.tif]

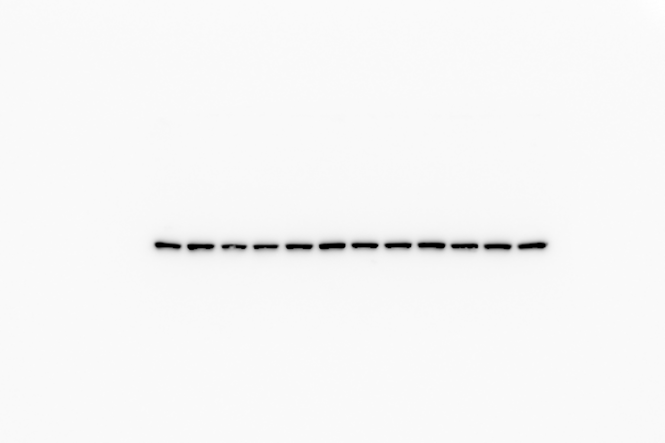

Supplement: Supplementary file 1 [file ijms-21-04588-s001.zip › Supplemental figs, table and data1/supplemental data 1_revise-2/S-22.tif]

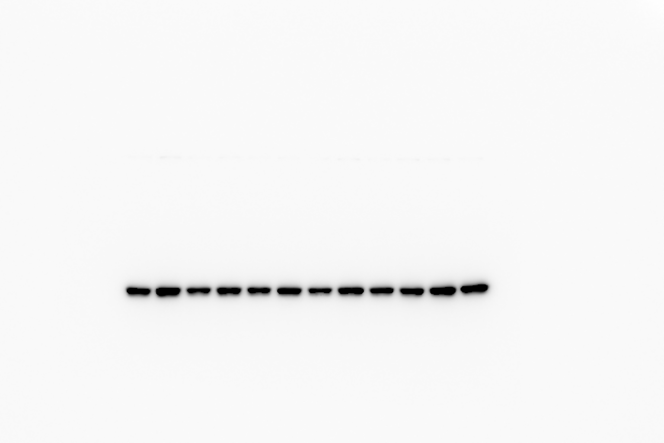

Supplement: Supplementary file 1 [file ijms-21-04588-s001.zip › Supplemental figs, table and data1/supplemental data 1_revise-2/S-23.tif]

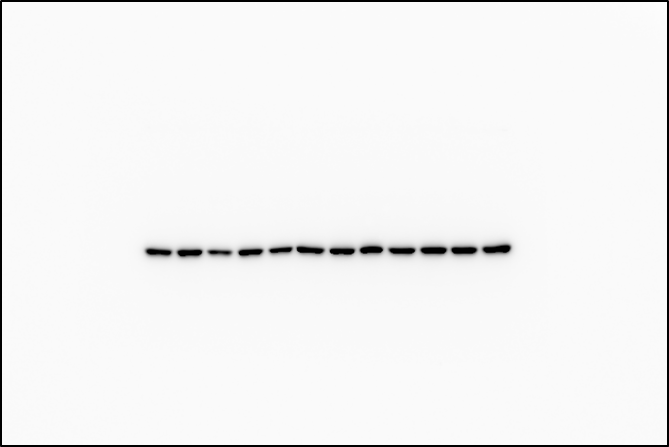

Supplement: Supplementary file 1 [file ijms-21-04588-s001.zip › Supplemental figs, table and data1/supplemental data 1_revise-2/S-24.tif]

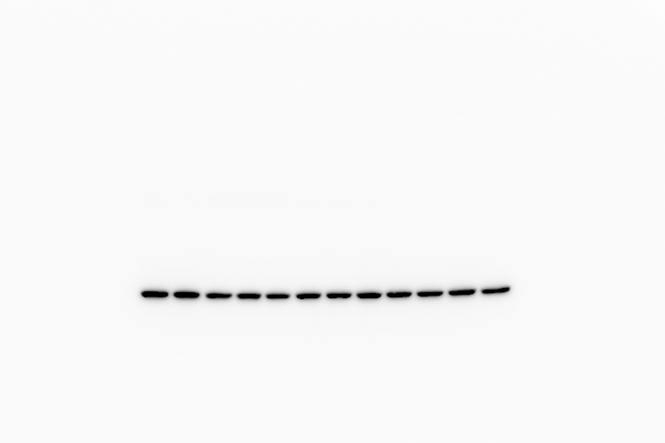

Supplement: Supplementary file 1 [file ijms-21-04588-s001.zip › Supplemental figs, table and data1/supplemental data 1_revise-2/S-25.tif]

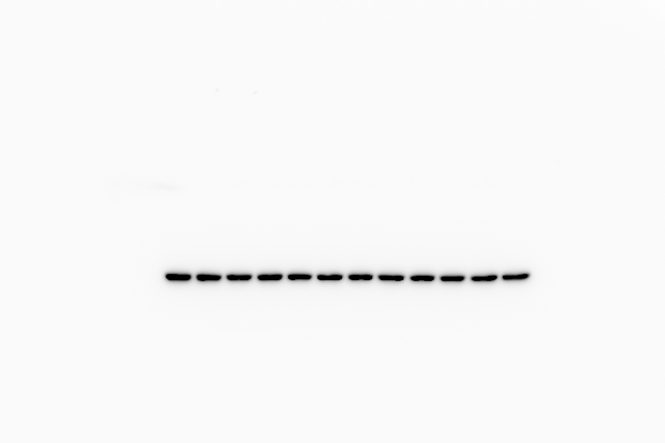

Supplement: Supplementary file 1 [file ijms-21-04588-s001.zip › Supplemental figs, table and data1/supplemental data 1_revise-2/S-26.tif]

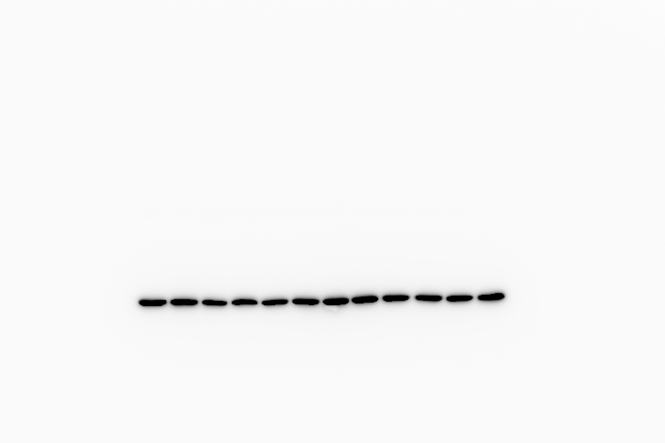

Supplement: Supplementary file 1 [file ijms-21-04588-s001.zip › Supplemental figs, table and data1/supplemental data 1_revise-2/S-27.tif]

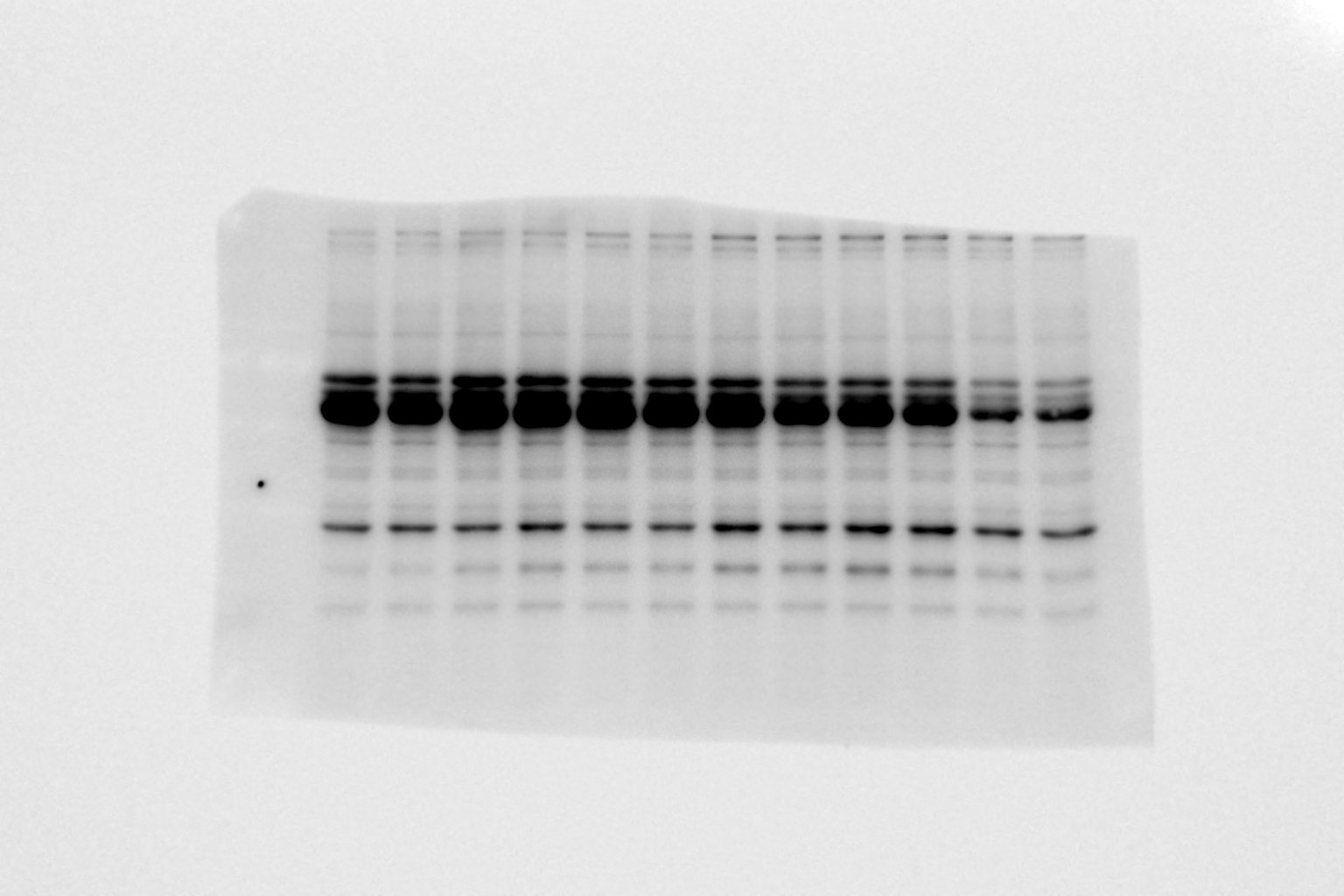

Supplement: Supplementary file 1 [file ijms-21-04588-s001.zip › Supplemental figs, table and data1/supplemental data 1_revise-2/S-28.tif]

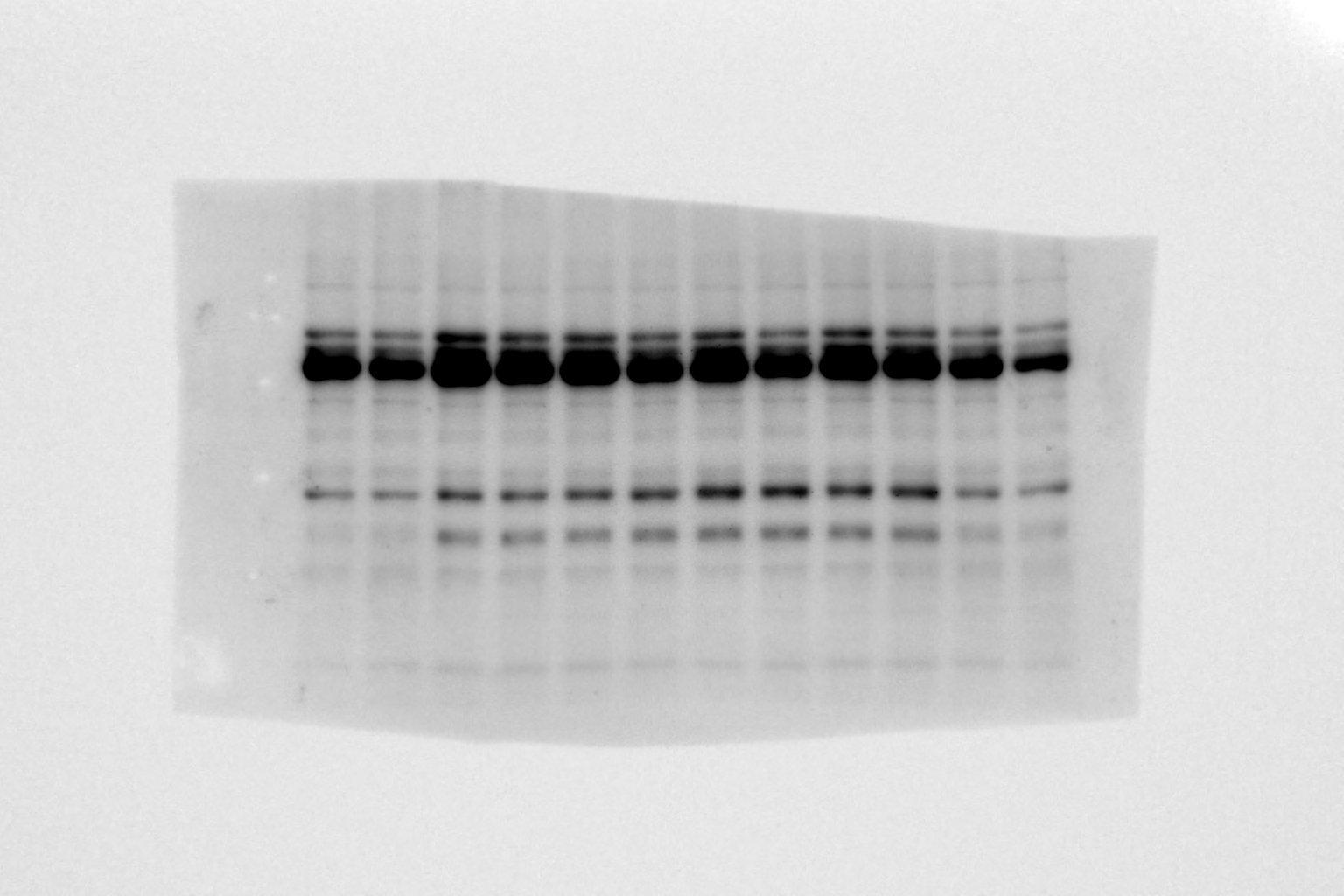

Supplement: Supplementary file 1 [file ijms-21-04588-s001.zip › Supplemental figs, table and data1/supplemental data 1_revise-2/S-29.tif]

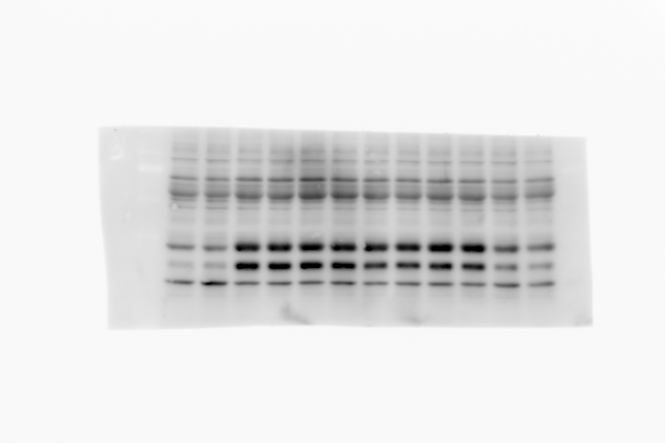

Supplement: Supplementary file 1 [file ijms-21-04588-s001.zip › Supplemental figs, table and data1/supplemental data 1_revise-2/S-3.tif]

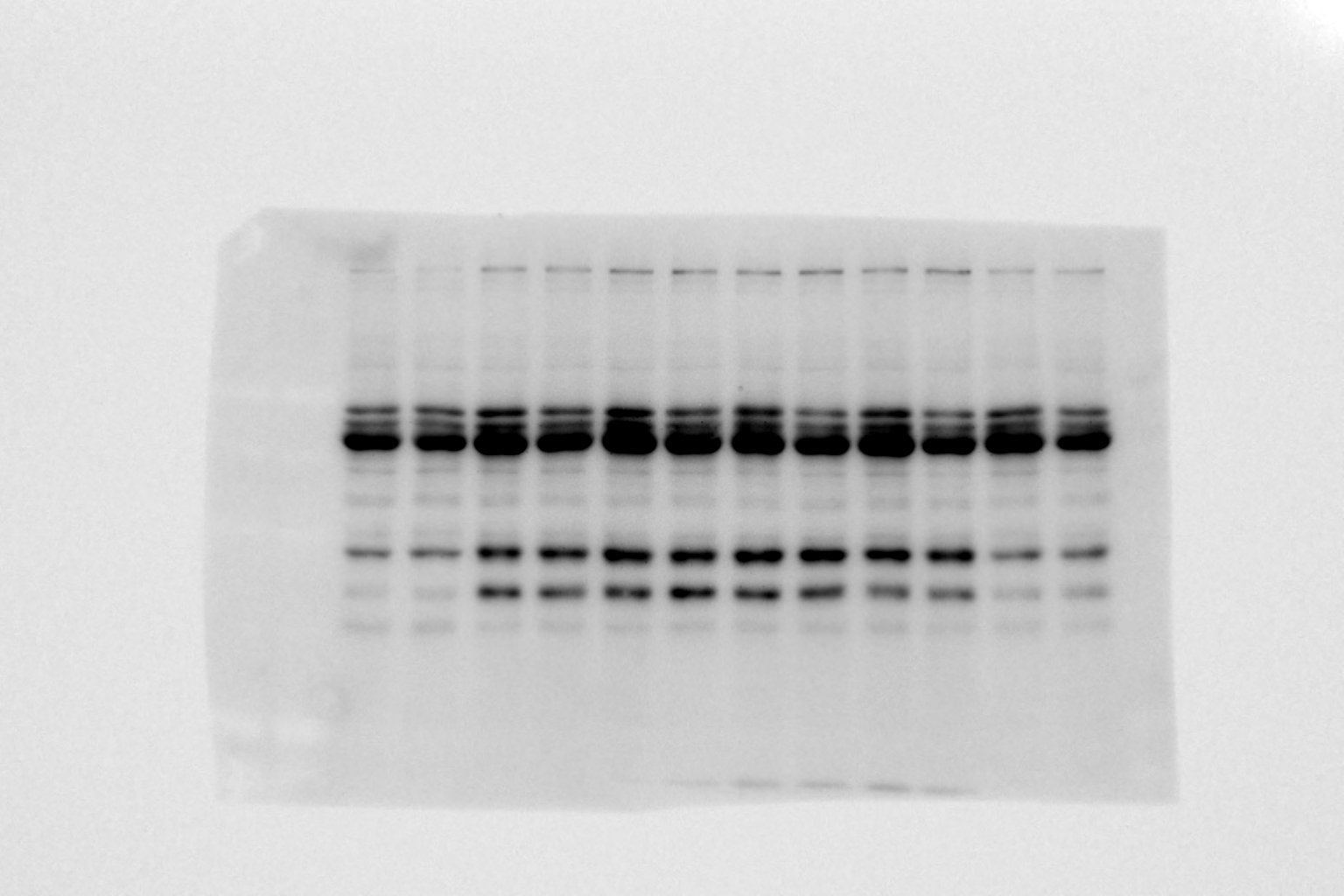

Supplement: Supplementary file 1 [file ijms-21-04588-s001.zip › Supplemental figs, table and data1/supplemental data 1_revise-2/S-30.tif]

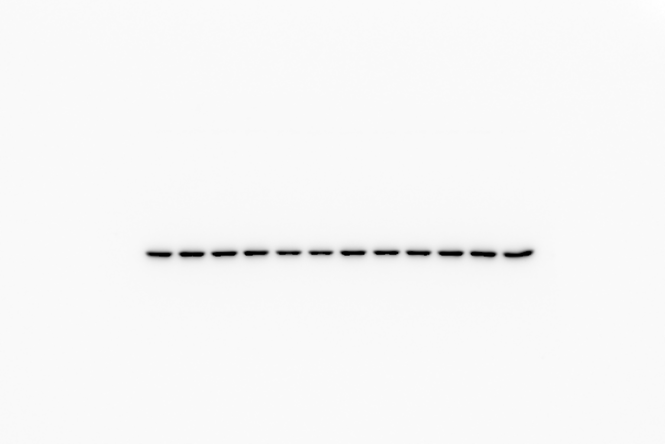

Supplement: Supplementary file 1 [file ijms-21-04588-s001.zip › Supplemental figs, table and data1/supplemental data 1_revise-2/S-31.tif]

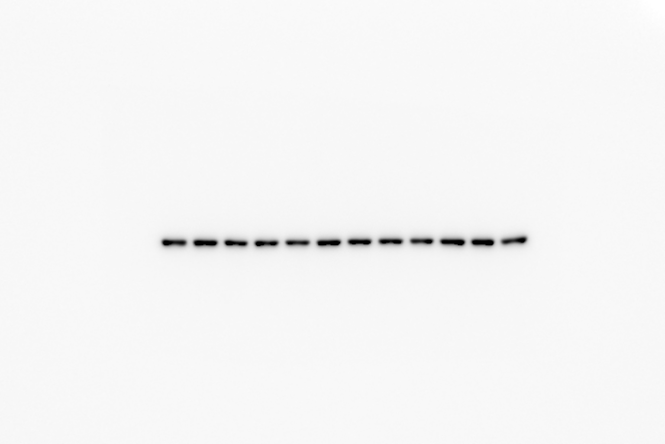

Supplement: Supplementary file 1 [file ijms-21-04588-s001.zip › Supplemental figs, table and data1/supplemental data 1_revise-2/S-32.tif]

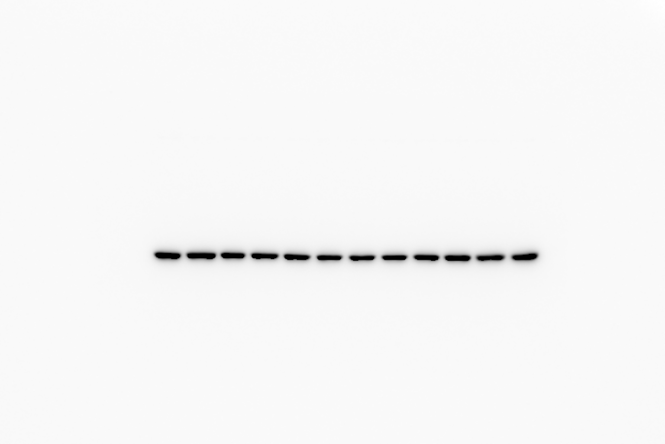

Supplement: Supplementary file 1 [file ijms-21-04588-s001.zip › Supplemental figs, table and data1/supplemental data 1_revise-2/S-33.tif]

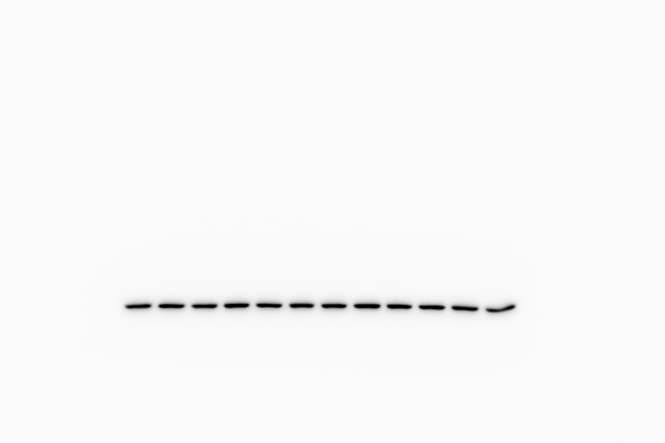

Supplement: Supplementary file 1 [file ijms-21-04588-s001.zip › Supplemental figs, table and data1/supplemental data 1_revise-2/S-34.tif]

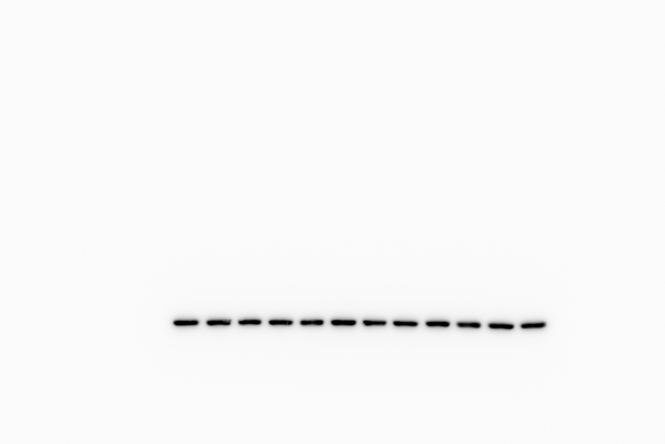

Supplement: Supplementary file 1 [file ijms-21-04588-s001.zip › Supplemental figs, table and data1/supplemental data 1_revise-2/S-35.tif]

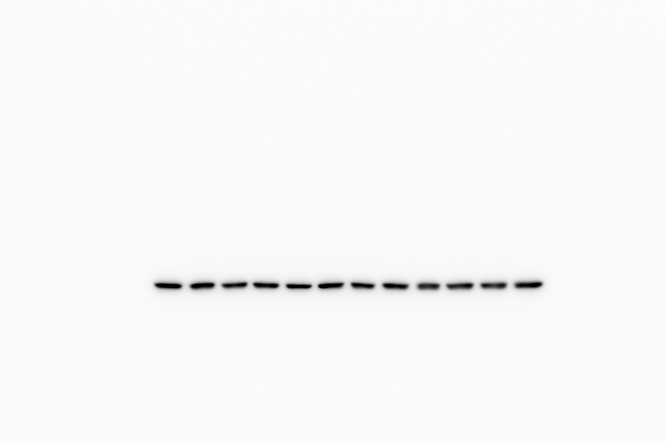

Supplement: Supplementary file 1 [file ijms-21-04588-s001.zip › Supplemental figs, table and data1/supplemental data 1_revise-2/S-36.tif]

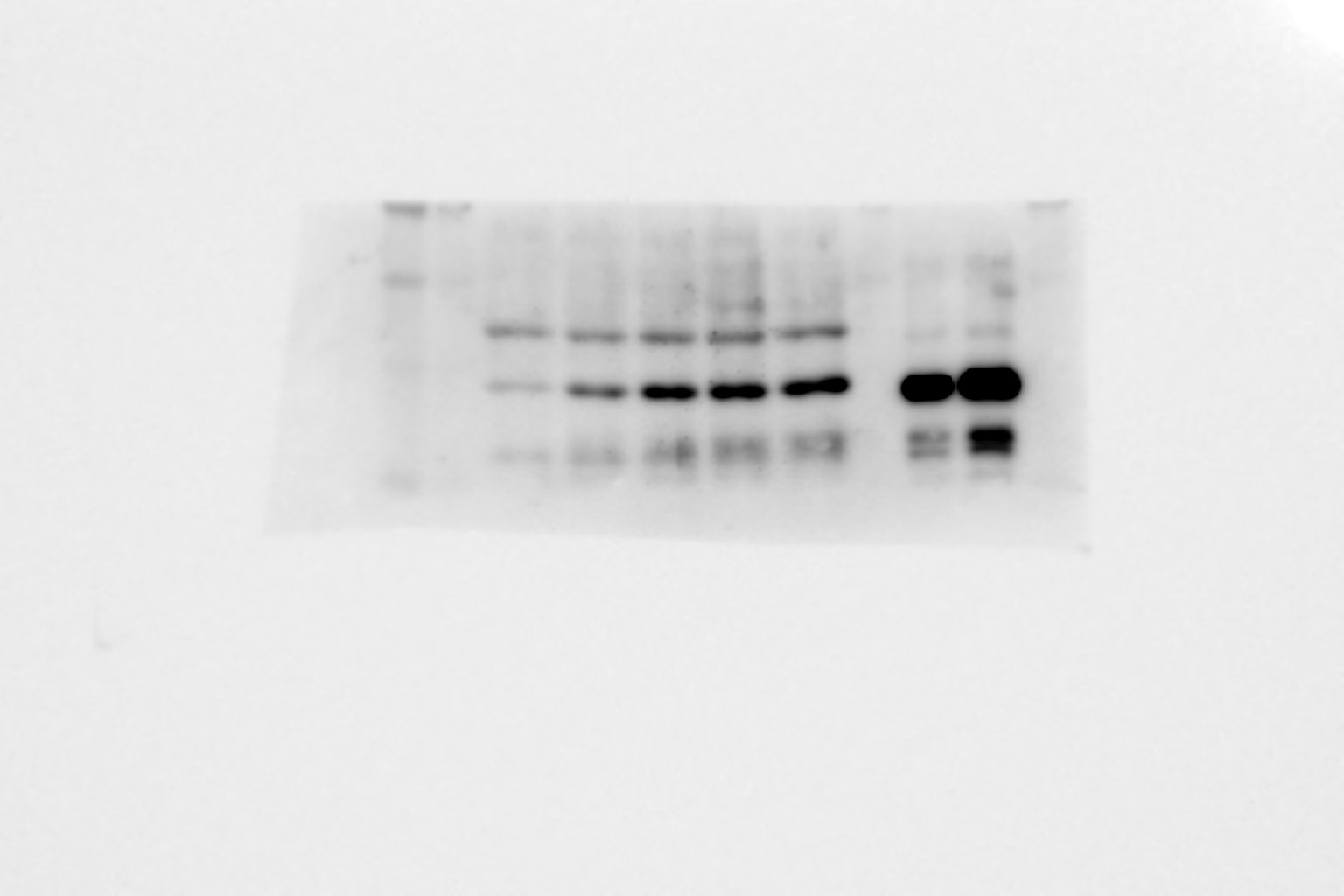

Supplement: Supplementary file 1 [file ijms-21-04588-s001.zip › Supplemental figs, table and data1/supplemental data 1_revise-2/S-37.tif]

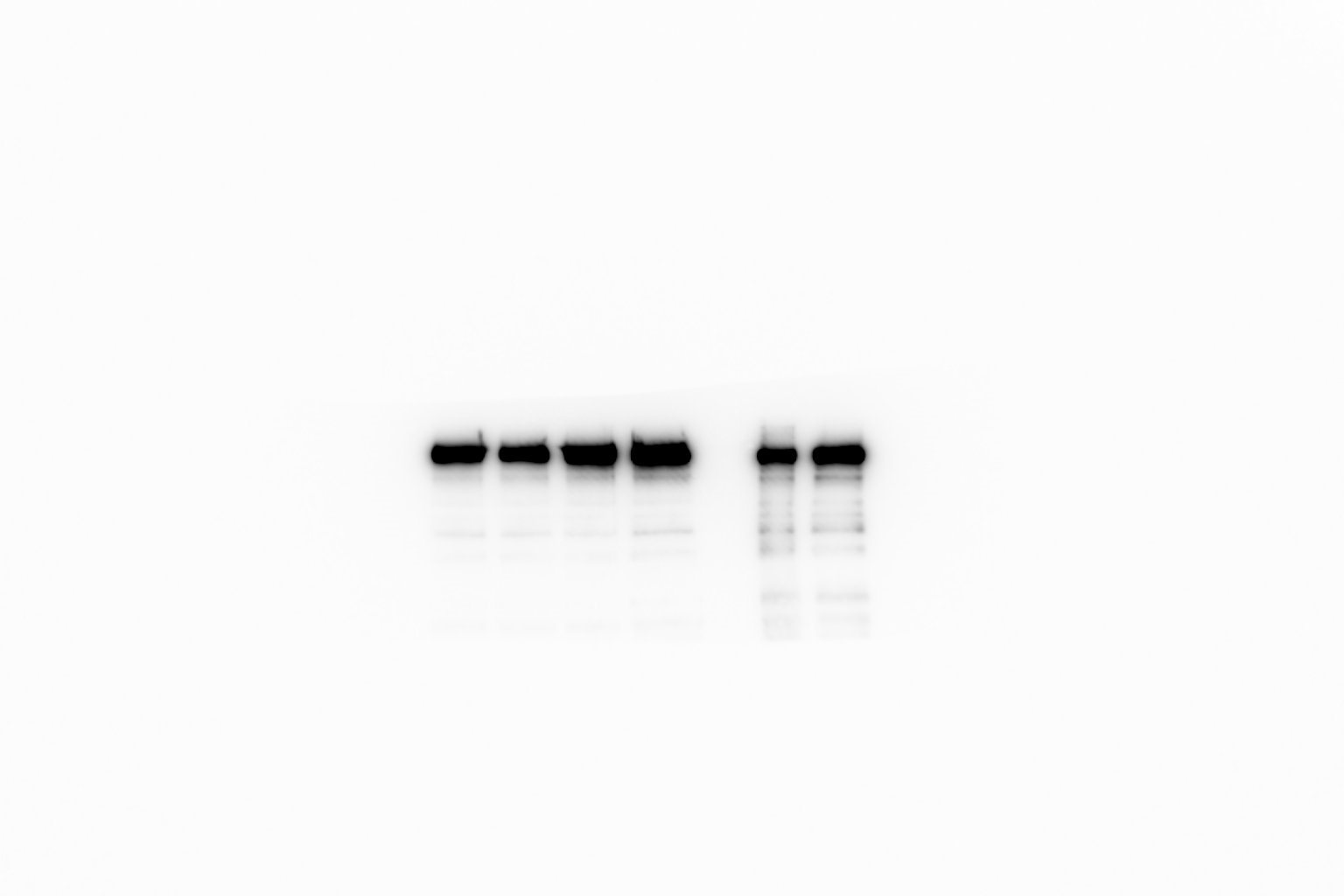

Supplement: Supplementary file 1 [file ijms-21-04588-s001.zip › Supplemental figs, table and data1/supplemental data 1_revise-2/S-38.tif]

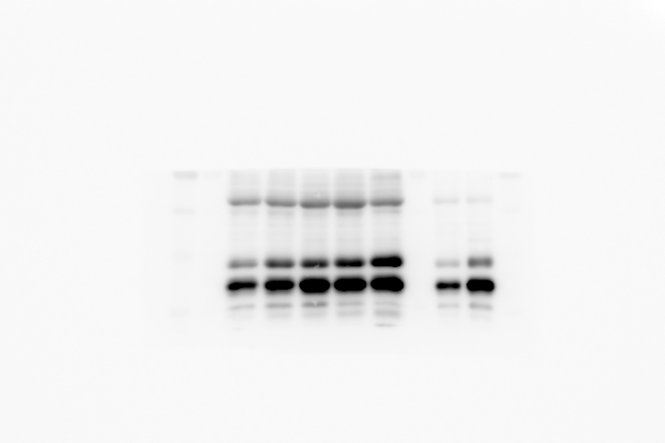

Supplement: Supplementary file 1 [file ijms-21-04588-s001.zip › Supplemental figs, table and data1/supplemental data 1_revise-2/S-39.tif]

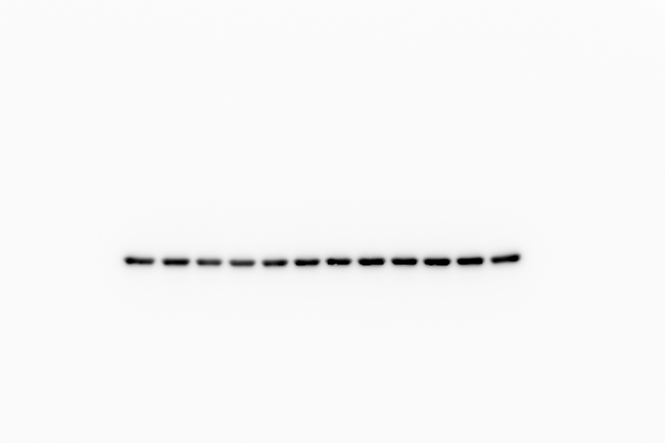

Supplement: Supplementary file 1 [file ijms-21-04588-s001.zip › Supplemental figs, table and data1/supplemental data 1_revise-2/S-4.tif]

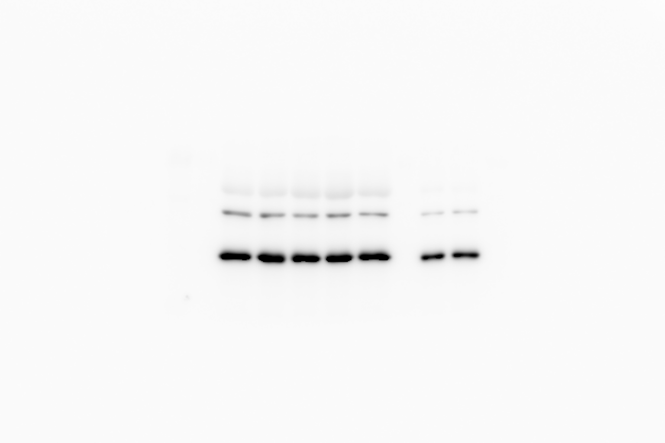

Supplement: Supplementary file 1 [file ijms-21-04588-s001.zip › Supplemental figs, table and data1/supplemental data 1_revise-2/S-40.tif]

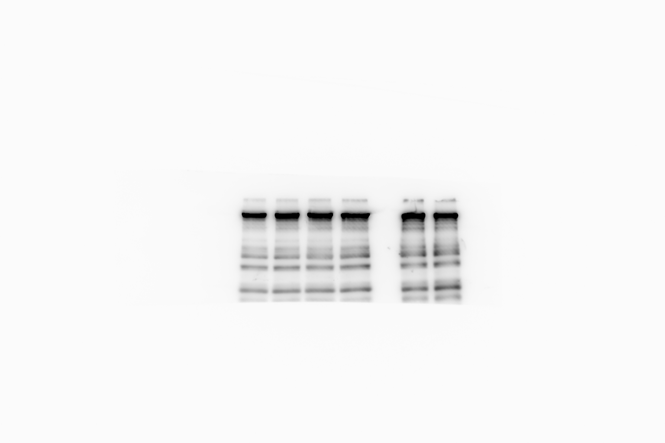

Supplement: Supplementary file 1 [file ijms-21-04588-s001.zip › Supplemental figs, table and data1/supplemental data 1_revise-2/S-41.tif]

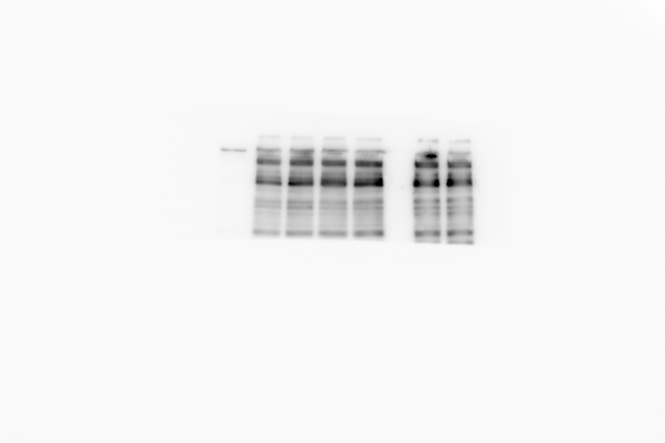

Supplement: Supplementary file 1 [file ijms-21-04588-s001.zip › Supplemental figs, table and data1/supplemental data 1_revise-2/S-42.tif]

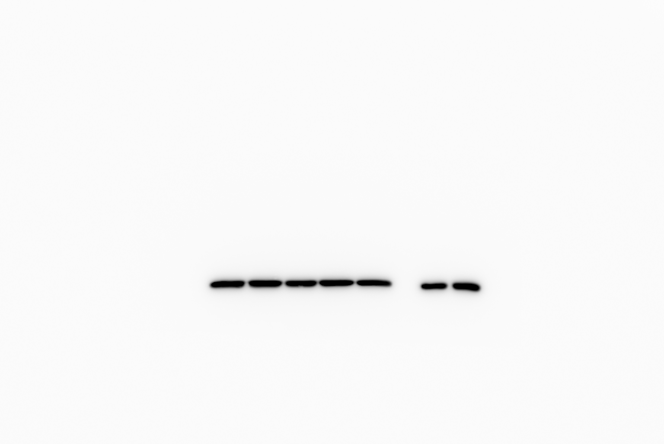

Supplement: Supplementary file 1 [file ijms-21-04588-s001.zip › Supplemental figs, table and data1/supplemental data 1_revise-2/S-43.tif]

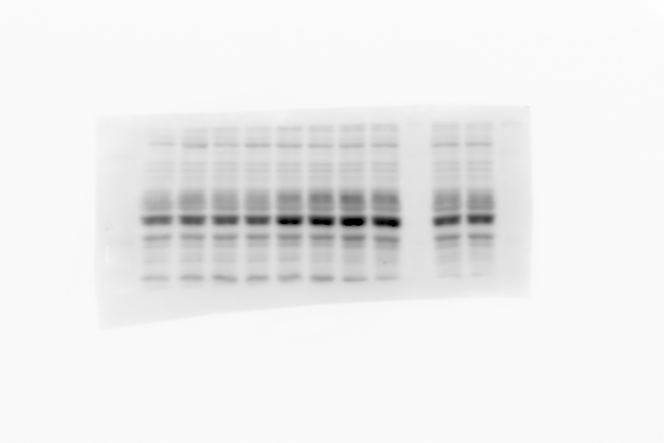

Supplement: Supplementary file 1 [file ijms-21-04588-s001.zip › Supplemental figs, table and data1/supplemental data 1_revise-2/S-44.tif]

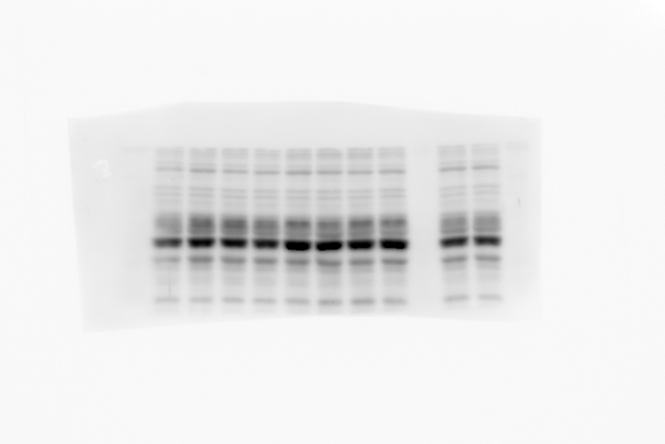

Supplement: Supplementary file 1 [file ijms-21-04588-s001.zip › Supplemental figs, table and data1/supplemental data 1_revise-2/S-45.tif]

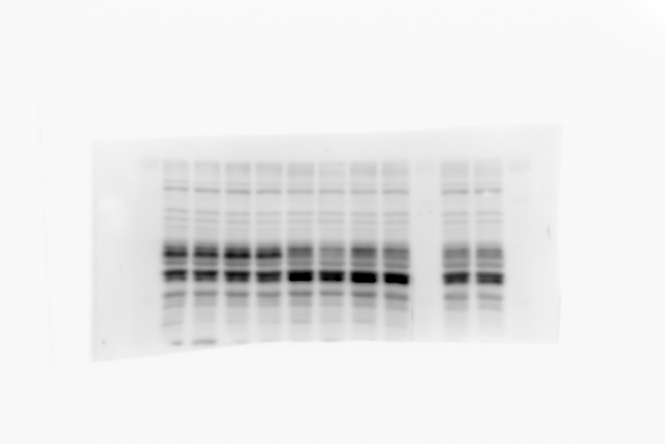

Supplement: Supplementary file 1 [file ijms-21-04588-s001.zip › Supplemental figs, table and data1/supplemental data 1_revise-2/S-46.tif]

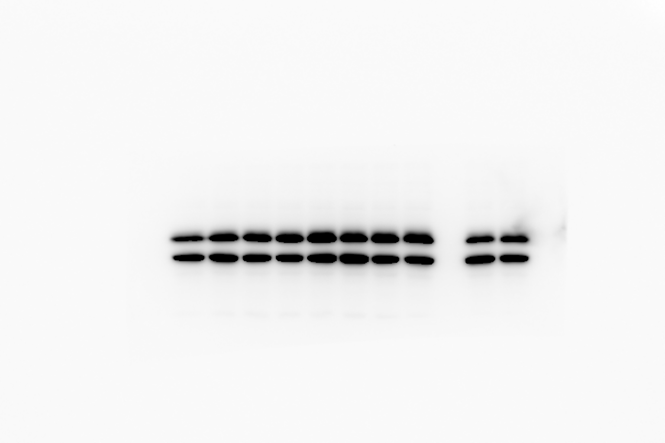

Supplement: Supplementary file 1 [file ijms-21-04588-s001.zip › Supplemental figs, table and data1/supplemental data 1_revise-2/S-47.tif]

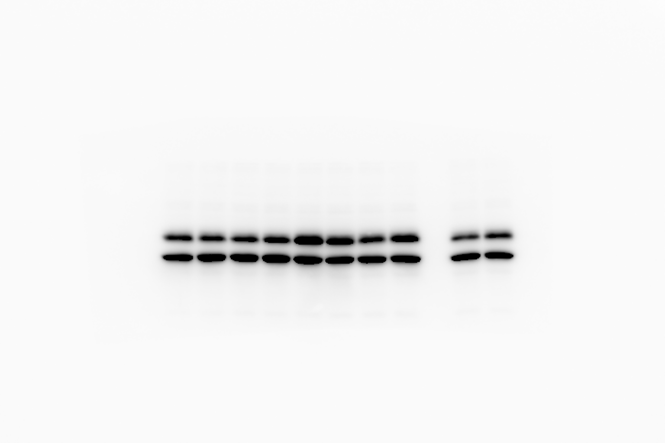

Supplement: Supplementary file 1 [file ijms-21-04588-s001.zip › Supplemental figs, table and data1/supplemental data 1_revise-2/S-48.tif]

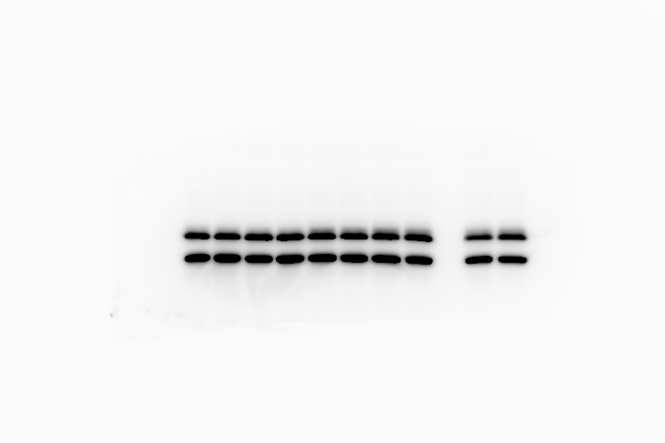

Supplement: Supplementary file 1 [file ijms-21-04588-s001.zip › Supplemental figs, table and data1/supplemental data 1_revise-2/S-49.tif]

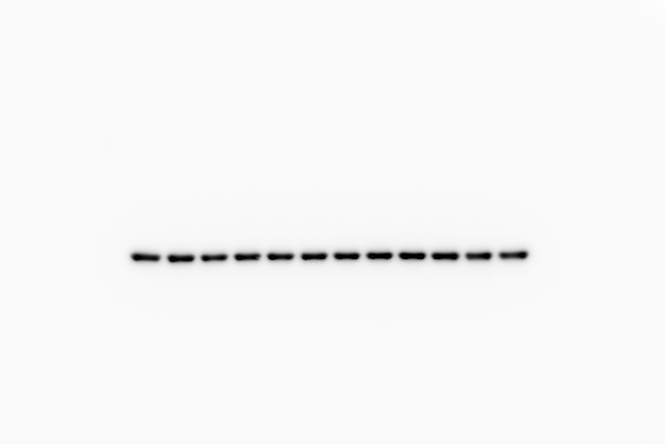

Supplement: Supplementary file 1 [file ijms-21-04588-s001.zip › Supplemental figs, table and data1/supplemental data 1_revise-2/S-5.tif]

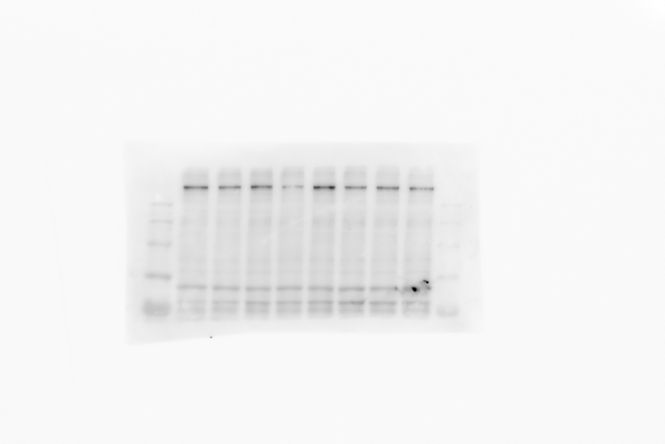

Supplement: Supplementary file 1 [file ijms-21-04588-s001.zip › Supplemental figs, table and data1/supplemental data 1_revise-2/S-50.tif]

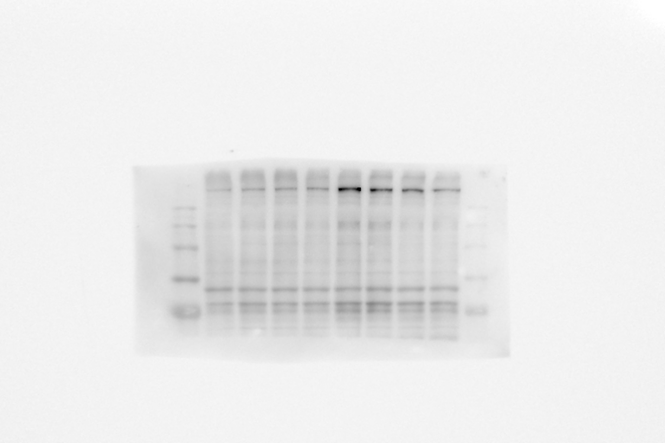

Supplement: Supplementary file 1 [file ijms-21-04588-s001.zip › Supplemental figs, table and data1/supplemental data 1_revise-2/S-51.tif]

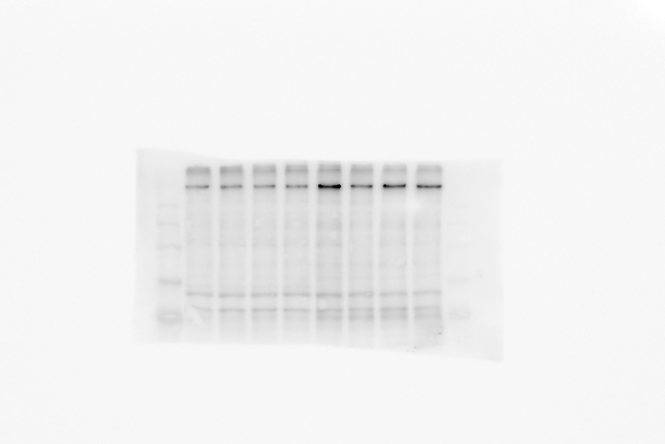

Supplement: Supplementary file 1 [file ijms-21-04588-s001.zip › Supplemental figs, table and data1/supplemental data 1_revise-2/S-52.tif]

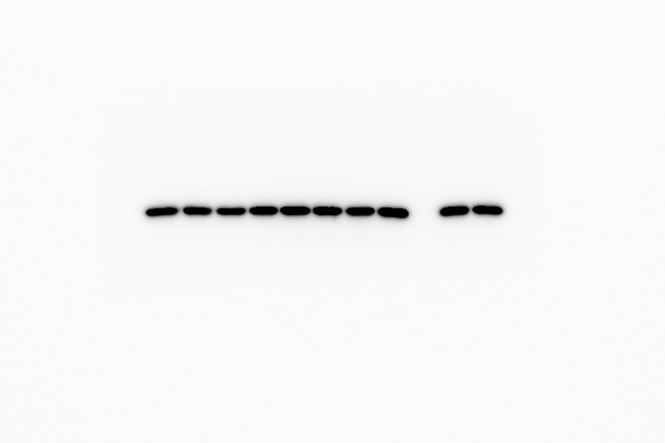

Supplement: Supplementary file 1 [file ijms-21-04588-s001.zip › Supplemental figs, table and data1/supplemental data 1_revise-2/S-53.tif]

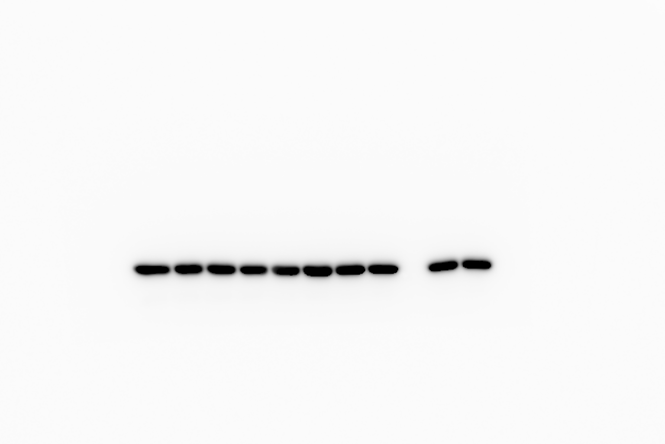

Supplement: Supplementary file 1 [file ijms-21-04588-s001.zip › Supplemental figs, table and data1/supplemental data 1_revise-2/S-54.tif]

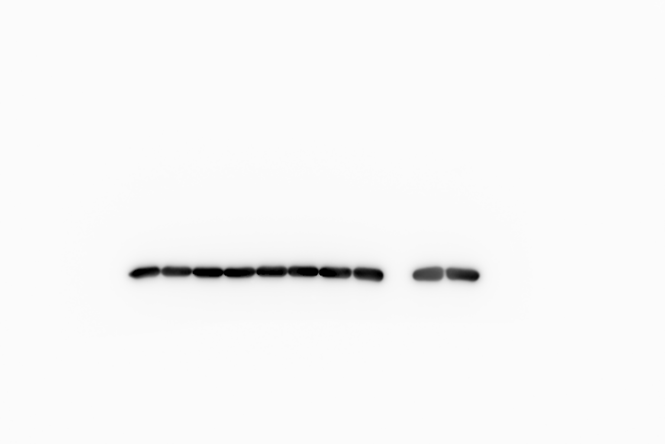

Supplement: Supplementary file 1 [file ijms-21-04588-s001.zip › Supplemental figs, table and data1/supplemental data 1_revise-2/S-55.tif]

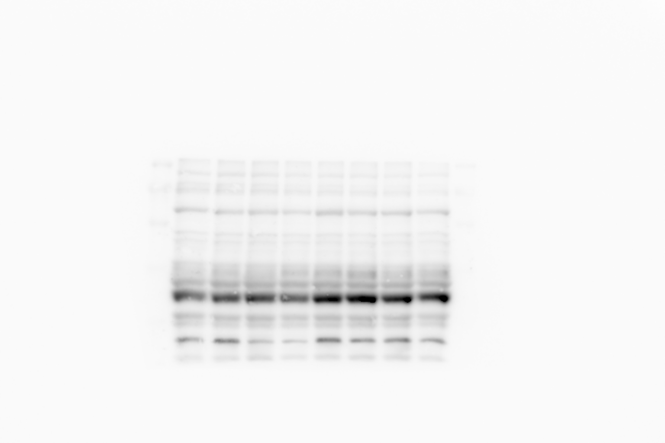

Supplement: Supplementary file 1 [file ijms-21-04588-s001.zip › Supplemental figs, table and data1/supplemental data 1_revise-2/S-56.tif]

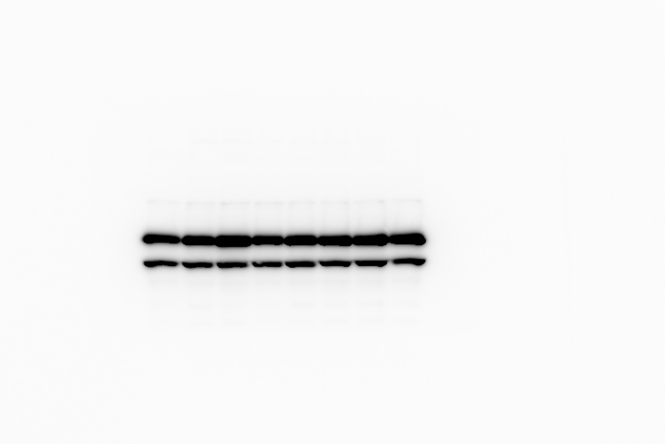

Supplement: Supplementary file 1 [file ijms-21-04588-s001.zip › Supplemental figs, table and data1/supplemental data 1_revise-2/S-57.tif]

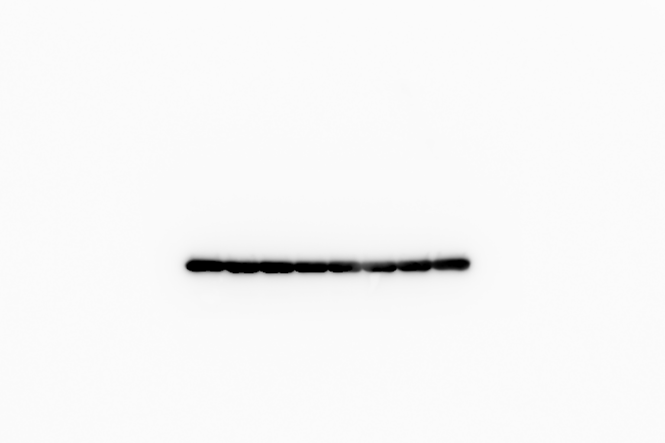

Supplement: Supplementary file 1 [file ijms-21-04588-s001.zip › Supplemental figs, table and data1/supplemental data 1_revise-2/s-58.tif]

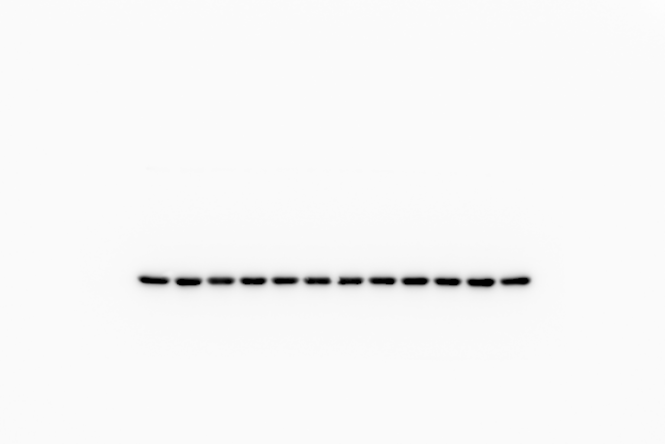

Supplement: Supplementary file 1 [file ijms-21-04588-s001.zip › Supplemental figs, table and data1/supplemental data 1_revise-2/S-6.tif]

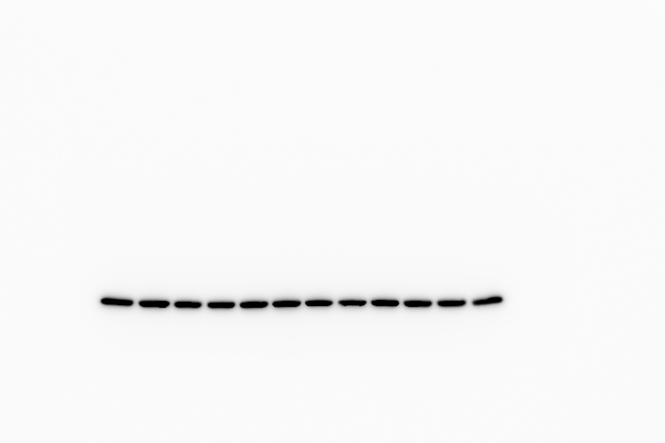

Supplement: Supplementary file 1 [file ijms-21-04588-s001.zip › Supplemental figs, table and data1/supplemental data 1_revise-2/S-7.tif]

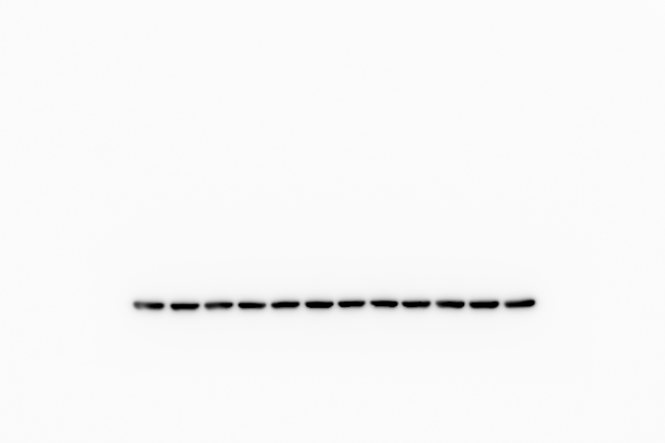

Supplement: Supplementary file 1 [file ijms-21-04588-s001.zip › Supplemental figs, table and data1/supplemental data 1_revise-2/S-8.tif]

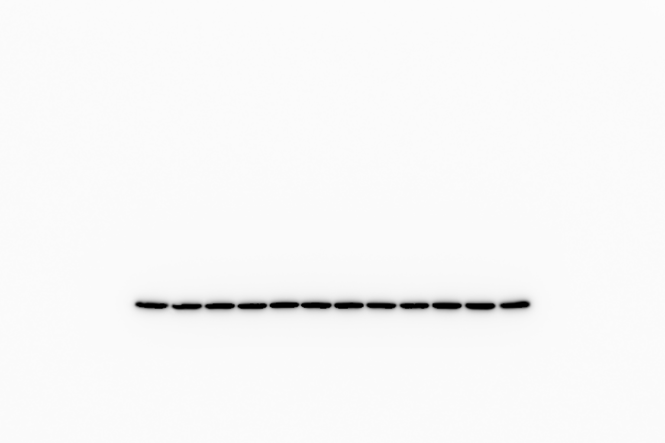

Supplement: Supplementary file 1 [file ijms-21-04588-s001.zip › Supplemental figs, table and data1/supplemental data 1_revise-2/S-9.tif]
